# Surviving COVID-19 and Battling Fibrosis: A Retrospective Cohort Study Across Three Pandemic Waves

**DOI:** 10.3390/diagnostics14242811

**Published:** 2024-12-13

**Authors:** Mihai Lazar, Ecaterina Constanta Barbu, Cristina Emilia Chitu, Mihaela Buzoianu, Andreea Catalina Petre, Catalin Tiliscan, Stefan Sorin Arama, Victoria Arama, Daniela Adriana Ion, Mihaela Cristina Olariu

**Affiliations:** 1 Faculty of Medicine, University of Medicine and Pharmacy Carol Davila, No. 37, Dionisie Lupu Street, Sector 2, 020021 Bucharest, Romania; mihai.lazar@umfcd.ro (M.L.); cristina.chitu@umfcd.ro (C.E.C.); catalin.tiliscan@umfcd.ro (C.T.); sorin.arama@umfcd.ro (S.S.A.); victoria.arama@umfcd.ro (V.A.); daniela.ion@umfcd.ro (D.A.I.); mihaela.olariu@umfcd.ro (M.C.O.); 2 National Institute for Infectious Diseases Prof. Dr. Matei Bals, No. 1, Calistrat Grozovici Street, Sector 2, 021105 Bucharest, Romania; mihaela.buzoianu@yahoo.com

**Keywords:** SARS-CoV-2, COVID-19, pneumonia, lung fibrosis, long-COVID

## Abstract

Background/Objectives: We aimed to characterize the fibrosis following COVID-19 pneumonia, using quantitative analysis, after three months and subsequently, after two years of patients’ release from the hospital, and to identify the risk factors for pulmonary fibrosis. Methods: We performed a retrospective, observational cohort study on 420 patients with severe forms of COVID-19. For all patients, we registered demographic, inflammatory and biochemical parameters, complete blood count and D-dimers; all patients underwent three computed tomography scans (at admittance, at 3 months and at 2 years). Results: We found fibrosis in 67.9% of patients at the 3-month evaluation and in 42.4% of patients at the 2-year evaluation, registering a significant decrease in the severe and moderate fibrosis cases, with a slight increase in the mild fibrosis cases. The risk of fibrosis was found to be proportional to the values of age, duration of hospital stay, inflammatory markers (ESR, fibrinogen), cytolytic markers (LDH, AST) and D-dimers. The highest correlations with lung fibrosis were registered for interstitial pulmonary involvement (for the 3-month evaluation) and total pulmonary involvement (for the 2-year evaluation). Conclusions: Lung fibrosis represents a significant post-COVID-19 complication found in 42% of patients with severe forms of pneumonia at the 2-year evaluation. A significant overall decrease in the severity of lung fibrosis was registered at the 2-year evaluation compared to the 3-month evaluation. We consider that the amount of interstitial pulmonary involvement represents the optimal parameter to estimate the risk of lung fibrosis following SARS-CoV-2 pneumonia.

## 1. Introduction

The COVID-19 pandemic, caused by the severe acute respiratory syndrome coronavirus 2 (SARS-CoV-2), has left significant and long-lasting effects on global health. Acute lung injury may be associated with severe complications, such as acute respiratory distress syndrome (ARDS) and pulmonary thromboembolism. Tracheal lesions and pneumomediastinum, with incidences reported as high as 13%, are also common acute complications of COVID-19, even in patients who have not undergone mechanical ventilation. These complications are linked to the fibrous-hyaline degeneration of the tracheal rings, mediated by the sonic hedgehog (SHH) and Wnt5a signaling pathways [1].

Beyond the acute infection, a range of post-viral complications has emerged, one of the most concerning being post-COVID-19 pulmonary fibrosis, associated with long-term respiratory complications, reduced lung function and a diminished quality of life.

Fibrotic changes in various organs (heart, kidney, lung, liver, et al.) are frequently associated with an inflammatory process, depending on the pathogen and the intensity of the inflammation [2,3,4,5,6]. Lung fibrosis following COVID-19 develops due to a complex disruption of homeostasis, involving inflammation, oxidative stress, coagulation abnormalities, chemoattractant mediators and key cytokines, such as TGF-β1 (transforming growth factor beta 1), PDGF (platelet-derived growth factor), IL (interleukin)-6, IL-11, and IL-17, which collectively drive the proinflammatory and profibrotic processes [7,8]. The immune response generated in COVID-19 patients ranges from mild forms to acute respiratory distress syndrome (ARDS) in severe cases, triggering a cascade of inflammatory changes, including the activation of fibroblasts, which can continue to produce collagen, leading to persistent fibrosis [7,9,10].

Not every individual who contracts COVID-19 will develop fibrosis. The current data include several risk factors that increase the likelihood of developing post-COVID-19 fibrosis. The severity of the initial infection is one of the most significant factors. Patients who experienced severe pneumonia, required mechanical ventilation, or developed ARDS are at a much higher risk of fibrosis. These patients are typically those who spent long periods in intensive care units (ICUs) and had prolonged exposure to high levels of oxygen therapy, which can also contribute to lung injury [10,11].

Some authors suggest that combining clinical parameters and quantitative CT scores with laboratory inflammatory markers—such as elevated IL-6 levels, C-reactive protein (CRP), and procalcitonin—could be an effective strategy for predicting the rapid progression to severe COVID-19 pneumonia. This approach may enhance therapeutic decision-making. Among the quantitative CT COVID scores, Quantitative Mean Density (QMD) has been reported to have the highest predictive power for identifying rapid progression [12].

Pre-existing conditions are another important consideration. Individuals with underlying chronic respiratory diseases, such as chronic obstructive pulmonary disease (COPD) or idiopathic pulmonary fibrosis (IPF), are more vulnerable to post-COVID-19 fibrosis [13]. Additionally, advanced age and comorbidities like diabetes, obesity, and cardiovascular disease are associated with worse COVID-19 outcomes and a higher risk of fibrotic complications [7].

Genetic predisposition may also play a role in determining who develops fibrosis. Studies have suggested that certain genetic markers related to the regulation of the immune system and fibrotic response could increase the susceptibility of certain individuals to develop pulmonary fibrosis [14]. Lung fibrosis may also occur following viral infections with the human T-cell leukemia virus (HTLV), human immunodeficiency virus (HIV), cytomegalovirus (CMV), Epstein–Barr virus (EBV), murine γ-herpesvirus 68 (MHV-68), influenza virus, avian influenza virus, Middle East respiratory syndrome (MERS)-CoV, severe acute respiratory syndrome (SARS)-CoV and SARS-CoV-2 [15].

Imaging studies are essential for diagnosing pulmonary fibrosis, with computerized tomography (CT) representing the main tool for diagnosing interstitial lung fibrosis according to the ATS/ERS/JRS/ALAT statement [16]. Lung CT scans can reveal characteristic features of fibrosis, such as ground-glass opacities, reticular patterns, traction bronchiectasis and bronchiolectasis, and honeycombing, which are indicative of scarring in the lungs [17,18]. The literature indicates that the most common patterns observed include linear bands, followed by ground-glass opacities and reticulations, predominantly in the posterior lung segments of both the upper and lower lobes. A reticular pattern with posterior distribution, distinct from other fibrotic changes identified in post-COVID-19 evaluations, has been associated with prolonged low-dose corticosteroid use. Additionally, bronchiectasis and volume loss have also been reported during short-term follow-ups after severe SARS-CoV-2 pneumonia [19].

Pulmonary function tests (PFTs) are also useful as they can quantify the extent of lung damage by measuring parameters like forced vital capacity (FVC) and diffusion capacity of the lungs for carbon monoxide (DLCO), which tend to be reduced in patients with fibrosis [20]. Several studies have observed that a mild reduction in DLCO, reported in up to 40% of patients, is the most common abnormality identified in respiratory function tests conducted during COVID-19 follow-up examinations [19].

The long-term outlook for patients with post-COVID-19 fibrosis is still uncertain. While some patients may experience the stabilization of their condition, others may continue to experience progressive lung function decline, potentially leading to severe respiratory failure. The variable nature of fibrosis in these patients underscores the need for ongoing research to better understand the mechanisms driving fibrosis, the associated risk factors and to identify effective treatments.

In this article, we aimed to characterize pulmonary fibrosis following the first three waves of COVID-19 using quantitative CT analyses conducted at 3 months and 2 years after discharge. Additionally, we sought to identify risk factors/predictors for pulmonary fibrosis in patients with the SARS-CoV-2 infection; based on them, we developed two fibrosis prediction models—one for fibrosis at 3 months and another for fibrosis at 2 years post-COVID-19. To the best of our knowledge, this is the first longitudinal study to evaluate both short-term (3 months) and long-term (2 years) post-COVID-19 lung fibrosis across the first three pandemic waves.

## 2. Materials and Methods

### 2.1. Study Population

This was a single-center retrospective, observational cohort study on 420 patients with severe forms of COVID-19 admitted to a tertiary hospital, exclusively dedicated to COVID-19 patients, between March 2020 and December 2023. The patients were divided into three groups based on hospital admittance date (corresponding to the first three COVID-19 waves): Group 1 (March 2020 to January 2021), Group 2 (February 2021 to June 2021) and Group 3 (July 2021 to December 2021).

We included adult patients (≥18 years old), with confirmed COVID-19 infections diagnosed by a positive real-time polymerase chain reaction (RT-PCR) test or SARS-CoV-2 rapid antigen test, with severe forms of COVID-19, with CT scans performed at admittance, and follow-up CT scans at 3 months, and, respectively, at 2 years after hospital leave, with a CT image quality score 4 or 5.

We excluded patients with (a) an age under 18, (b) pregnancy, (c) prior known chronic pulmonary diseases: chronic obstructive pulmonary disease, chronic bronchitis, pulmonary emphysema, pulmonary tuberculosis, bronchiectasis, (d) idiopathic interstitial lung disease, (e) rheumatic diseases (rheumatoid arthritis, Sjögren syndrome, systemic lupus erythematosus, systemic sclerosis, etc.), (f) smoking, toxic environmental exposures, (g) chronic treatment with methotrexate, amiodarone, (h) the presence of severe gastro-esophageal reflux documented before admittance, (i) an image quality score of 1 to 3 for the CT scan; (j) invasive ventilation; and (k) pneumonia episodes during the follow-up period (between the 3-month evaluation and the 2-year evaluation) [18]. 

### 2.2. Definitions

We considered a severe form of COVID-19 to be when the patients presented at least one of the following criteria: peripheral oxygen saturation (SpO_2_) ≤ 93% in the ambient air, respiratory rate (RR) > 30/min, arterial oxygen partial pressure to fractional inspired oxygen ratio (PaO_2_/FiO_2_ ratio) < 300 or lung infiltrates > 50% of lung parenchyma [21].

The overall image quality of each CT scan was evaluated based on the image definitions of lung parenchyma, anatomic contours of vessels and mediastinum and the presence of breathing or motion artifacts:

1. Poor image definition, poor defined contour, important motions artifacts due to breathing or movement;
2. Acceptable image definition, acceptable defined contours, moderate motions artifacts due to breathing or movement;
3. Acceptable image definition, acceptable defined contours, sparse motions artifacts due to breathing or movement;
4. Good image definition, good defined contours, minimal motions artifacts due to breathing or movement;
5. Excellent image definition, excellent defined contours no artifacts due to breathing or movement.

The patients were considered to have fibrosis if a specific pattern was observed on CT images performed at the 3-month or 2-year follow-up evaluations: ground-glass, linear, reticular, honeycomb and bronchiectasis.

### 2.3. Demographic and Biological Parameters

We registered, for all patients included in our study, demographic parameters (sex, age, duration of hospital stay), inflammatory markers (C reactive protein (CRP), fibrinogen, serum ferritin, erythrocyte sedimentation rate (ESR), IL-1 and IL-6), biochemical parameters (alanine aminotransferase, creatin-kinase, lactate dehydrogenase), complete blood count (erythrocytes, leukocytes, lymphocytes, neutrophils) and D-dimers.

### 2.4. CT Examination Protocol

All patients were evaluated by CT scans with a 64-slice Definition AS (Siemens Healthcare GmbH, Munich, Germany) at hospital admission, at the 3-month, and, respectively, 2-year follow-ups, after their release from the hospital. All examinations were performed in helical mode with CAREDose4D, and CARE kV was activated to reduce the radiation dose. The acquisition parameters are presented in Table 1.

For the quantitative analysis of lung lesions, we used syngoPulmo3D (syngo.via VA30B), which allowed for the measurement of the percentage and volume of lung lesions according to a defined density range scale.

The imaging evaluation was blinded; the radiologists were unaware of the identity of the patients in the study groups for all three CT scans (performed at admission date, and at the 3-month and 2-year follow-ups). They were also not informed which study group the patients were enrolled in.

### 2.5. Fibrosis Analysis

Two radiologists (LM, BM), with 22 and 15 years of experience in radiology, respectively, assessed the lung CT scans through consensus reading, focusing on image quality, parenchymal changes and quantitative evaluation.

The initial qualitative evaluation of lung fibrosis involved identifying specific fibrosis patterns on the obtained CT images, including ground-glass, linear, reticular, honeycomb, and traction bronchiectasis. For each patient, all visible fibrosis patterns on the scanned images were documented during both the 3-month and 2-year evaluations.

For the quantitative evaluation, we ensured operator-independent, reproducible measurements of fibrosis using a dedicated software for lung volumetric evaluation, syngoPulmo3D (Siemens Healthcare, syngo.via VA30B). This software enables the automatic segmentation of the lungs, followed by the calculation of lung volume based on specific density ranges and vessel subtraction [18].

To assess the extent of lung injury at admission, we used the following density range scale for the CT images:

- Higher than 0 HU—alveolar lesions (consolidations);
- Between 0 and −200 HU—mixed lesions (alveolar and interstitial);
- Between −201 and −800 HU—interstitial lesions;
- Between −801 and −1000 HU—normal lung parenchyma [18].

Pulmonary fibrosis at the 3-month and 2-year follow-ups was quantified by measuring the percentage of lung volume with densities between −201 and −800 HU on the CT scans for each patient (a method also used in our previous work) [18].

We categorized fibrosis severity as follows:

- Mild fibrosis: lung involvement between 1% and 9%;
- Moderate fibrosis: lung involvement between 9% and 20%;
- Severe fibrosis: lung involvement exceeding 20%.

### 2.6. Identification of the Risk Factors/Predictors for Pulmonary Fibrosis in Patients with SARS-CoV-2 Infection

The association between fibrosis and the patient data recorded at admission was evaluated using Spearman’s correlation, while the odds ratio (OR) was determined through logistic regression analysis.

Receiver operating characteristic (ROC) curve analyses was conducted for all explanatory variables predicting lung fibrosis individually to compare their performance. The optimal cutoff point was determined by identifying the point on the ROC curve farthest from the diagonal (Youden’s J).

A multivariable logistic regression analysis was conducted to further explore the relationship between fibrosis and different parameters, leading to the development of fibrosis prognostic models [18].

### 2.7. Statistical Analysis

Statistical analysis was conducted using the Statistical Package for Social Sciences (SPSS, version 25, IBM Corp., Armonk, NY, USA). Continuous variables are presented as medians with quartiles (Q1, Q3), while categorical variables are expressed as percentages. To compare the three groups, an ANOVA with a post hoc LSD test was applied. Spearman’s correlation, odds ratio (OR), and receiver operating characteristic (ROC) curve analyses were utilized to identify parameters associated with lung fibrosis. Multivariable logistic regression was used to develop a fibrosis prognostic model. The logistic regression was performed using a backward elimination approach, where independent variables were excluded based on the Wald test results for individual parameters from the logistic regression analysis. Variables with the least significant effect (*p* > 0.2) were removed from the prognostic model. The overall significance of the regression model was assessed using the Omnibus test of model coefficients, with a *p*-value of less than 0.05 considered as statistically significant [18].

## 3. Results

### 3.1. Demographic, Biological and Radiological Parameters at Admission

Our study included 60.71% males (255 patients) and 39.29% females (165 patients), registering a male-to-female sex ratio of 1.54 to 1, with a median age of 52 [43; 63] years for the male patients and 54 [42; 62.5] years for the female patients. The hospitalization period varied between 1 and 64 days with a median value of 11 days, without significant differences between the three groups of patients. The duration of antiviral treatment and corticotherapy were also similar in all three groups.

The median values for all of the patients at admission in the hospital are presented in Table 2, stratified into three groups.

We found an increased ESR in 80.96% of patients, CRP in 76.8%, fibrinogen in 63.5%, and serum ferritin in 72.2% of patients, with the median values presented in Table 2. Comparing the inflammatory markers in the three groups, we found slightly increased values for the patients in Group 3.

The patients also presented increased plasmatic aminotransferases in 32.7% of cases, LDH in 44.8% of cases and creatin kinase in 24.3% of cases, also with slightly increased values for the patients in Group 3. In the post hoc analysis between groups, we found variations with statistical significance for the parameters mentioned above, only for the patients in Group 2 (Table 3), showing less severe changes, with no significant variation between the patients in Groups 1 and 3.

The blood-work analysis revealed variations in the leukocytes count in 33.8% of patients (decreased values in 23.3% and increased values in 10.5%), in the neutrophils count in 37.9% of patients (decreased values in 9.8% and increased values in 28.1%), and lymphopenia in 54.1% of patients, with no significant variation between the three groups. 

We registered lower plasmatic values for erythrocytes in 34.7% of patients and for platelets in 28.5% of patients, with statistical significance for the patients in Group 3 compared to the patients in Group 1 and Group 2. 

D-dimers were increased in 28.7% of patients with no variation, with statistical signification between the study groups.

The extent of lung involvement at hospital admission, evaluated by CT scans, is presented in Table 4.

We registered a median lung involvement of 35.5% of the lung volume, representing mainly interstitial lesions (31.5%), with higher values for the patients in Group 3 and a less severe pulmonary involvement for the patients in Group 2 (Table 3).

In the post hoc analysis between groups, presented in Table 5, we found variations with statistical significance for the number of “pulmonary lobes with pneumonia” and the “interstitial involvement” measured in Group 2 patients compared to those in Groups 1 and 3, consistent with the less severe lung involvement for the patients included in Group 2. The median values for lung involvement in Group 3 are slightly higher than in Group 1, although no statistically significant variance was registered between both groups.

### 3.2. Quantification of Pulmonary Fibrosis at the 3-Month and 2-Year Follow-Ups

In our research, pulmonary fibrosis was found as follows: at the 3-month evaluation, we detected it in 67.9% of patients, while at the 2-year evaluation, we registered it in 42.4% of them. Regarding the fibrosis grades detected, we obtained a significant decrease in the severe fibrosis cases (from 11.6% to 0%) and in the moderate fibrosis cases (from 21.2% to 1.2%), with a slight increase in the mild fibrosis cases (from 35% to 41.2%). This evolution pattern was found in all three groups except Group 2, where we found, at the 2-year evaluation, a decrease in all types of fibrosis, including mild fibrosis.

A complete regression of fibrosis was observed in 107 patients (37.5% from the fibrosis cases recorded at the 3-month evaluation), stratified as follows: 33 patients (33.6%) in Group 1, 43 patients (52.4% from the fibrosis cases recorded at the 3-month evaluation) in Group 2, and 31 patients (29.5% from the fibrosis cases recorded at the 3-month evaluation) in Group 3.

The most frequent fibrosis pattern found was the “ground-glass” at the 3-month evaluation (61.9% of patients) and the linear/reticular pattern at the 2-year evaluation (39.7% of patients) (Table 6).

All patients exhibiting a honeycomb pattern of fibrosis at the 3-month evaluation maintained this pattern at the 2-year evaluation. The honeycomb pattern was the only fibrosis type where no regression in the number of affected patients was observed over the 2-year follow-up period. 

The fibrotic lesions were mainly distributed subpleurally in mild fibrosis, with the involvement of the other lung areas in the moderate and severe cases. 

The stratification of the three groups showed a higher fibrotic involvement in Groups 1 and 3 (in both the 3-month and 2-year evaluations) than in Group 2. 

### 3.3. Risk Factors/Predictors for Pulmonary Fibrosis

We further evaluated the correlations between the registered parameters presented in Section 3.1 and lung fibrosis at 3 months and at 2 years, respectively.

The risk of fibrosis in both timelines (3 months and 2 years, respectively) was found to be proportional to the values of age, duration of hospital stay, inflammatory markers (ESR, fibrinogen), cytolytic markers (LDH, AST) and D-dimers. Among the demographic parameters, the highest association with fibrosis was observed for hospital stay (0.447 at 3 months and 0.241 at 2 years). Regarding inflammatory markers, ESR showed the strongest association (0.451 at 3 months and 0.233 at 2 years), while for biochemical markers, LDH exhibited the highest values (0.392 at 3 months and 0.248 at 2 years). In our research, fibrosis significantly correlated with all imaging parameters, registering the highest correlation for the interstitial pulmonary involvement (0.732 for the 3-month evaluation) and for the total pulmonary involvement (0.476 for the 2-year evaluation) (Table 7).

**Figure 1:**
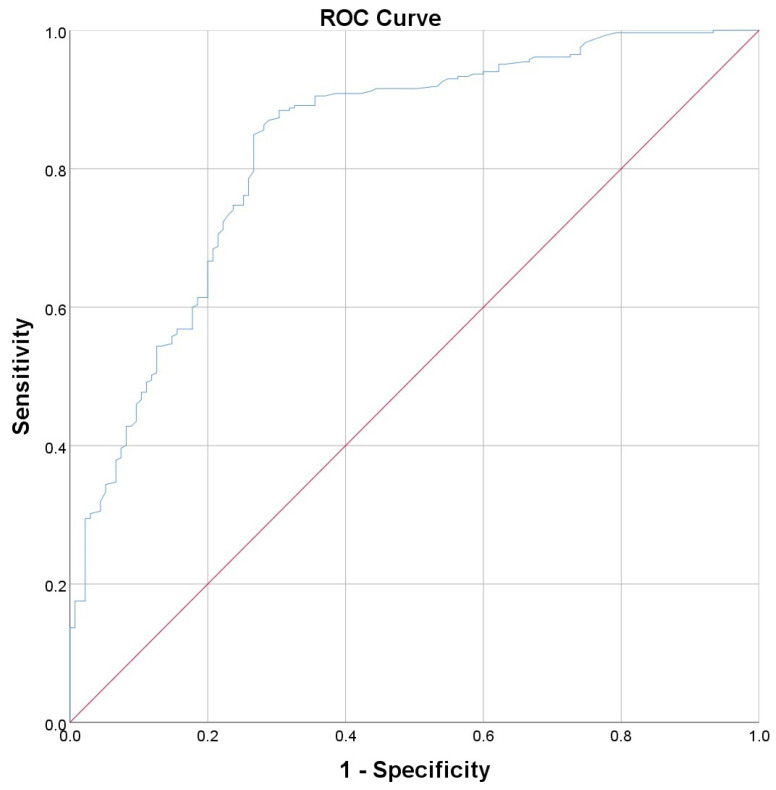
Receiver operating characteristics (ROC) curve for the ability of the “interstitial pulmonary involvement” to predict pulmonary fibrosis at 3 months. The area under the curve (AUC) = 0.831 (0.788−0.873, CI 95%).

**Figure 2:**
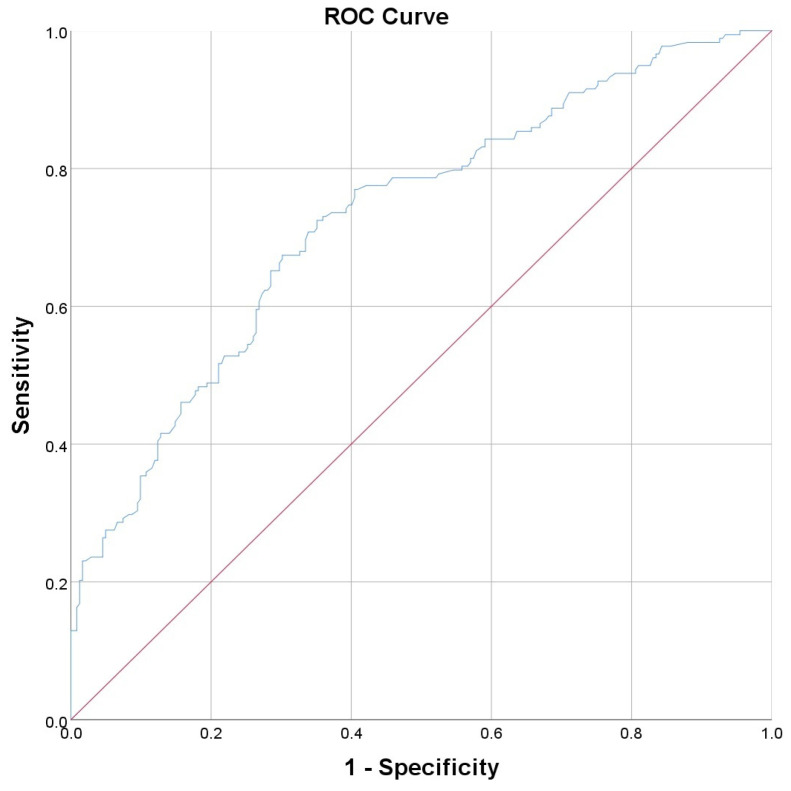
Receiver operating characteristics (ROC) curve for the ability of the “interstitial pulmonary involvement” to predict pulmonary fibrosis at 2 years. The area under the curve (AUC) = 0.728 (0.679−0.777, CI 95%).

To compare the performance of the individual parameters from Table 7 presenting a significant association with lung fibrosis at both the 3-month and 2-year evaluations, we performed ROC curves (Table 8, Figure 1 and Figure 2). The ROC curves for all 16 parameters listed in Table 8 are provided as Supplementary Material.

For the 3-month fibrosis, we obtained the highest AUC for the parameter “interstitial pulmonary involvement” (Figure 1), while for the 2-year fibrosis, we obtained similar values for “interstitial pulmonary involvement” (Figure 2) and “total pulmonary involvement”. Therefore, we consider that the amount of interstitial pulmonary involvement represents the optimal parameter to estimate the risk of lung fibrosis following SARS-CoV-2 pneumonia.

Calculating Youden’s J in the case of “interstitial pulmonary involvement”, we obtained a value of 25.25% for the interstitial pulmonary involvement, which can estimate the occurrence of fibrosis at 3 months with a sensitivity (Se) of 0.85 and a specificity (Sp) of 0.73. In the case of the 2-year fibrosis, the optimal cutoff point for the parameter “interstitial pulmonary involvement” was 31.7%, which can predict the occurrence of fibrosis with a Se of 0.71 and a Sp of 0.66. The specificity to predict lung fibrosis increases with the percentage of interstitial lung involvement, with a specificity of 100%, for the patients with over 57% “interstitial pulmonary involvement”, to present fibrosis at both the 3-month and 2-year evaluations.

To improve the risk analysis for post-COVID-19 lung fibrosis, we performed a multivariable logistic regression analysis using all of the registered parameters presented in Table 2. The results are presented in Table 9, for the fibrosis at the 3-month evaluation, and in Table 10, for the fibrosis at the 2-year evaluation.

Based on the data in Table 8, we can also calculate the probability of interstitial fibrosis at three months using the following formula: EXP (Constant + 0.026 × CRP + 0.009 × LDH − 0.031 × AST + 1670 × Lymphocytes + 0.084 × Interstitial pulmonary involvement + 0.048 × hospital stay)/[1 + EXP (Constant + 0.026 × CRP + 0.009 × LDH − 0.031 × AST + 1670 × Lymphocytes + 0.084 × Interstitial pulmonary involvement + 0.048 × hospital stay)]. The Omnibus tests of the model coefficients for the model presented above were lower than 0.001, registering an overall accuracy prediction of 85.7% for 3-month fibrosis.

Considering the data presented in Table 9, the probability of interstitial fibrosis at two years can be calculated using the following formula:

EXP (Constant + 0.412 × Lymphocytes − 0.059 × Duration of corticotherapy + 0.612 × Pulmonary lobes with pneumonia + 0.062 × Interstitial pulmonary involvement)/[1 + EXP (Constant + 0.412 × Lymphocytes − 0.059 × Duration of corticotherapy + 0.612 × Pulmonary lobes with pneumonia + 0.062 × Interstitial pulmonary involvement)]. The Omnibus tests of the model coefficients for the model presented above were lower than 0.001, registering an overall accuracy prediction of 70.1% for 2-year fibrosis.

## 4. Discussion

### 4.1. Post-COVID-19 Fibrosis Pathways

The pathways of fibrosis in the SARS-CoV-2 infection include hypoxemia-induced fibrosis, macrophage-induced fibrosis and viral–fibroblast interaction [7].

Hypoxemia-induced fibrosis. SARS-CoV-2 causes complex pathological mechanisms in the lungs that induce a decrease in gas exchanges and hypoxemia [22]. Thus, lung inflammation caused by SARS-CoV-2 leads to the diffuse impairment of alveoli (alveolar–capillary barrier disruption with oedema and exudate, loss of type II pneumocytes with reduced surfactant synthesis, fibrin deposition and formation of hyaline membranes, thickening of alveolar septa with fibroblasts and lymphomonocytic infiltrate, hyperplastic pneumocytes desquamation with intra-alveolar epithelial debris, interstitial fibrosis) and microthrombi [22,23]. In conditions of alveolar injury, hypoxemia and hypoxia of type II pneumocytes, there is an upregulation of hypoxia-inducible factor-1α (HIF-1α) and profibrotic genes, including for transforming growth factor beta-1 (TGF-β1) [7,24]. HIF-1α activates the production of inflammatory cytokines (including IL-1, IL-6, and TNF-α) and regulates viral replication of SARS-CoV-2 through its nucleocapsid (N) protein [25]. In our research, we found that IL- 1 presented higher values in the patients of Group 3 (the third wave), while IL-6 presented increased plasmatic values in all three groups. Aditionally, our analysis revealed that the increase in IL-6 at the 3-month evaluation represents a risk factor for lung fibrosis. The COVID-19-induced hypoxemia determines the increase in a “hypoxia-responsive protein” named galectin 1 (Gal-1), a lectin that plays an important role in pulmonary fibrosis [25]. The hypoxia of hyperplastic type II pneumocytes promotes the profibrotic activation of cells inside the lungs by the interaction of Gal-1 and focal adhesion kinase 1 (FAK 1); Gal-1 activates FAK 1 in pneumocytes and the activation of FAK/ERK/S100A4 initiates the migration, proliferation and transdifferentiation of fibroblasts into myofibroblast with the deposition of high quantities of collagen and α-smooth muscle actin (α-SMA), as fibrotic damage [7,24,26,27]. Additionally, TGF-β1 activates and increases the FAK 1 through the profibrotic pathway of Wnt/β-catenin [7,25]. Also, Gal-1 sustains the immunosuppresive role of IL-10 on macrophages, natural killer cells and on T lymphocytes, and it is proven to promote tissue injury into the lungs [28]. SARS-CoV-2 induces persistent inflammation in the lungs through the activation of the NLRP3 inflammasome. This activation occurs upon the virus entering epithelial alveolar cells, leading to their destruction, which further exacerbates lung damage by impairing gas exchange and resulting in secondary hypoxemia and fibrotic changes [23].

Macrophage-induced fibrosis. SARS-CoV2 induces the differentiation in population phenotypes of CD163/LGMNMφ macrophages that are involved in fibrotic damage by expressing genes such as TGF-β I, SPP1, CCL18 and LGMN (Legumain). Also, these types of macrophages interact with many other cell types, such as fibroblasts, myofibroblasts, and pericytes, with the release of collagen, fibroblast growth factor (FGF) and TGF-β1, among others and thus, promote fibrotic damage [29,30].

Viral–Fibroblast Interaction. SARS-CoV-2 presents tropism for many receptors as integrins αvβ3 and αvβ6, which promote fibroblast differentiation into myofibroblasts and epithelial–mesenchymal transition (EMT) mediated by TGF-β1 with consequent fibrosis [31,32]. The N protein of SARS-CoV-2 was proven to increase the α-SMA expression in human fibroblast line 1 (HFL-1) type cells, which act as myofibroblasts which release ECM proteins, such as collagen, TGF-β1, some types of matrix metalloproteinases (MMP) and tissue inhibitors of metalloproteinase (TIMP), and thus, promote lungs fibrosis [33,34]. Additionally, TGF-β1 has an important role in fibroblast migration, proliferation and differentiation into myofibroblasts with the secretion of ECM proteins, promoting fibrosis [35,36].

### 4.2. Risk Factors Associated with Post-COVID-19 Fibrosis 

Demographic parameters have played a critical role in understanding COVID-19’s impact on different populations. The published data indicate that approximately 20% of COVID-19 patients needed hospitalization, particularly the elderly and chronically unwell patients [37], with a higher risk for medium or severe forms of COVID-19 in midlife adults and older adults compared to young adults, the men being disproportionately affected by medium and severe courses of disease compared to women [38]. A meta-analysis conducted on patients with post-COVID-19 pulmonary fibrosis, that included 2018 participants, demonstrated that fibrotic lung abnormalities were significantly associated with an older age (mean age of 59 years was found for the fibrotic patients, while the non-fibrotic patients were much younger, with a mean age of 48.5 years). Conclusive explanations for this association have not yet been established, but it has been hypothesized that older patients are more susceptible to develop a severe form of both SARS and MERS that are similar to the SARS-CoV-2 infection, with these viruses sharing a significant genetic similarity [13,39]. In our study, we also found a higher percentage of males with severe forms of COVID-19 (60.71%) with a median age of 54 years, without significant differences between the first three waves of COVID-19, suggesting their increased risk for lung fibrosis [40]. Additionally, in our study, the risk of fibrosis in both timelines (3 months and 2 years) was also found to be proportional to the duration of hospital stay.

Inflammatory markers play a key role in assessing the severity and progression of COVID-19, with fibrinogen, CRP, ESR and serum ferritin being among the most significant indicators of inflammation, strongly associated with a hyperinflammatory state, indicating an evolution towards both immediate and long-term complications [41,42,43].

An excessive inflammatory response can trigger the important fibroblast lung activation, and the consecutive fibrosis [7]. In our study, both ESR and fibrinogen demonstrated a significant association with lung fibrosis at the 3-month and 2-year evaluations, with ESR showing higher correlation coefficients and AUC values compared to other inflammatory markers such as CRP, fibrinogen, ferritin, IL-1 and IL-6. Elevated plasmatic fibrinogen also decreases coagulation times, promoting microthrombi formation in the lungs, a pathological change also suggested by the increased plasmatic values of D-dimers (found in 28.7% of patients in our study). Increased levels of plasma D-dimers have been demonstrated at high percentages among COVID-19 patients (approximatively 50%), in correlation with disease severity [44], which has been established to have a significant impact on the development of pulmonary fibrosis following SARS-CoV-2 pneumonia. IL-6 is also associated with increasing fibrinogen synthesis and triggering the extrinsic pathway of coagulation, followed by tissue factor-mediated thrombin generation and a hyper-coagulable status, proven to be characteristic of the severe forms of SARS-CoV-2 pneumonia [45]. Impaired oxygen exchange, in the case of local thrombosis, induces local hypoxia and secondary lung fibrosis [7]. In our research, we found that IL-1 presented higher values in patients of Group 3 (the third wave), while IL-6 presented increased plasmatic values in all three groups. Aditionally, our analysis revealed that the increase in IL-6 at the 3-month evaluation represents a risk factor for lung fibrosis, while the risk of fibrosis in both timelines (3 months and 2 years) was found to also be proportional to the values of inflammatory markers (ESR and fibrinogen).

As for the biochemical findings correlated with a greater severity of COVID-19, increased serum LDH levels, lymphopenia and leukocytosis have been detected. The high level of serum LDH was found to be significantly correlated to the extent of lung involvement and the inflammatory response involving fibroblastic proliferation, with a greater risk of developing post-COVID-19 pulmonary fibrosis. In the present study, we also identified elevated serum LDH, as well as elevated cytolytic enzyme AST, as risk factors for pulmonary fibrosis following COVID-19 [46]. Elevated AST and ALT have been found in patients with more severe lung involvement, suggesting that higher levels of this enzyme may correlate with a greater risk of complications like acute respiratory distress syndrome (ARDS) and, eventually, post-COVID fibrosis, following the hyperinflammatory state [47].

Comparing the plasmatic values of the inflammatory and cytolytic markers between groups, we observed reduced variations between the patients in Groups 1 and 3, suggesting a similar degree of severity for the first and third COVID-19 waves, and a significant decrease in the inflammatory and cytolytic markers for the patients in Group 2, corresponding to a lower severity for the second COVID-19 wave.

Changes in lymphocyte and neutrophil counts, also found in our study, are critical markers of disease severity and can be linked to the development of lung fibrosis. Lymphopenia, or a significant reduction in lymphocytes, is commonly observed in severe cases of COVID-19, while neutrophil counts tend to rise, indicating an increased inflammation status [48]. Experimental studies have shown that alveolar damage alone is not enough to start the process of fibrosis. The presence of neutrophil extracellular traps (NETs) is also required and play a significant role in lung fibrosis during COVID-19 infection. In the inflammatory process, released cytokines also stimulate neutrophil degranulation and increase oxidative stress damage, which, in turn, increases lung fibrosis [7]. 

Patients with severe forms of the SARS-CoV-2 infection, who require ventilatory support, also present a higher risk of developing pulmonary fibrosis; the underlying mechanism of the fibrotic process is considered to be the local injury due to trauma from mechanical ventilation, thus leading to a release of proinflammatory cytokines, exacerbating the acute response and contributing to the onset of post-COVID-19 pulmonary fibrosis [49,50]. In our study, to better demonstrate the association between COVID-19 and lung fibrosis, we excluded the patients who required invasive ventilation, considering this factor to be an independent factor that can induce fibrosis, disregarding the etiology of lung inflammation.

According to the literature, patients with comorbidities, such as obesity, arterial hypertension, coronary artery disease or diabetes mellitus, develop more severe forms of SARS-CoV-2 infections, with a higher risk for lung fibrosis [51]. Lung fibrosis may increase vascular resistance, reduce pulmonary hematosis, alter alveolar gas exchange with persistent hypoxia, and further aggravate endogen dysfunctions [52]. 

Permanent lung fibrosis was found, by Han et al., to be associated with a more severe initial CT lung involvement [53]. Their findings were consistent with previous results of our work, where we demonstrated that, if quantified, the percentage of interstitial pulmonary lesions could represent a relevant imaging parameter to estimate the risk for pulmonary fibrosis at post-COVID-19 follow-ups [18].

### 4.3. Evaluation of Post-COVID-19 Fibrosis 

Computerized tomography (CT) evaluation is the primary tool for diagnosing lung fibrosis, according to the ATS/ERS/JRS/ALAT statement [16]. Pulmonary CT findings associated with pulmonary interstitial fibrosis include ground-glass opacities, reticular opacities, traction bronchiectasis, and honeycomb cysts [16].

According to the Fleischner Society, fibrosis severity can be measured using visual assessment or by analyzing CT data in a semi-automated manner; the visual estimation of fibrosis can be categorized as mild, moderate or severe, or can be expressed as the percentage of lungs affected, rounded to the nearest 5%, 10% or 25%, but this method relies heavily on the person performing the assessment [16,54]. Another approach involves dividing the lungs into upper, mid and lower zones, which can be applied to both interspaced and volumetric CT datasets [54]. While studies have shown high reproducibility among trained observers, there is still variability in visual CT assessment due to differences between observers. The quantitative image analysis of lung fibrosis is a rapidly evolving field and can be valuable for accurately evaluating the extent of fibrosis [54]. Our study addresses these concerns and provides both a reproducible quantitative evaluation of lung fibrosis and also, at the same time, a qualitative evaluation of lung fibrosis patterns.

In severe COVID-19 pneumonia, CT scans often reveal extensive areas of ground-glass opacities, consolidation, and sometimes crazy-paving patterns, indicating widespread alveolar damage and inflammation, findings that are more pronounced in patients with severe forms of pneumonia [55]. Patients with greater lung involvement on CT scans are at a higher risk of developing post-COVID-19 pulmonary fibrosis. Persistent inflammatory responses in the lung, as seen in severe cases, can trigger fibroblast activation and the excessive deposition of collagen, leading to scarring of the lung tissue [9]. Post-COVID fibrosis, at 3–24 months after the SARS-CoV-2 infection, was described on CT scans in various percentages ranging from 9% [56] to 84%, depending on the severity of lung involvement [43]. For patients with severe forms of pneumonia and a lung involvement of over 40% (similar to the median lung involvement in our study), the communicated level of fibrosis is approximately 55% [57]. In our study, we found fibrosis in 67.9% of patients at the 3-month evaluation and in 42.4% of patients at the 2-year follow-up evaluation, with a significant decrease in the severe and moderate fibrosis cases and a slight increase in the mild fibrosis cases, demonstrating a partial reversibility of the post-COVID-19 fibrotic process, depending on the severity of lung changes.

The most frequent fibrosis pattern found in our study was the “ground-glass” at the 3-month evaluation and the linear/reticular pattern at the 2-year evaluation, with a mainly subpleural distribution of the fibrotic lesions in mild fibrosis, and involvement of the central lung areas in moderate and severe cases. Lung involvement was higher for the patients in the first and third COVID-19 waves compared to the second wave, a fact also consistent with the registered inflammatory changes. 

Considering the results of our study, we recommend the extent of “interstitial pulmonary involvement” to be a predictor for both short-term (3 months) and long-term (2 years) post-COVID-19 fibrosis, using the cut-off values of 25.5% for 3-month and 31.7% for 2-year evaluations; for the patients with over 57% “interstitial pulmonary involvement”, the risk of presenting fibrosis at both the 3-month and 2-year evaluations has a specificity of 100%.

The long-term lung changes following COVID-19 infection are still not completely clarified, and further studies are required. Therefore, it is mandatory for patients to have a follow-up after COVID-19 diagnosis, especially for those with associated risk factors for post-COVID-19 pulmonary fibrosis.

Study limitations: Lung fibrosis was diagnosed and assessed exclusively via CT scans; no lung function tests were performed to further characterize interstitial lung fibrosis. Patients with pre-existing conditions associated with lung fibrosis were excluded from the study to ensure a clearer evaluation of whether SARS-CoV-2 alone could induce lung fibrosis. In such cases, fibrosis following COVID-19 may exhibit a different progression compared to the patterns observed in our research. This study focused on patients with severe forms of COVID-19, and therefore, does not reflect the prevalence of lung fibrosis in the general COVID-19 population, which is expected to be lower at both the 3-month and 2-year evaluations due to the proportion of mild and moderate cases.

## 5. Conclusions

To the best of our knowledge, this is the first longitudinal study to evaluate both short-term (3 months) and long-term (2 years) post-COVID-19 lung fibrosis across the first three pandemic waves. 

The patients in Groups 1 and 3 (corresponding to the first and third waves of COVID-19) presented more pronounced changes in inflammatory markers, cytolytic parameters, and lung involvement, compared to the patients in Group 2 (corresponding to the second wave of COVID-19).

Lung fibrosis represents a significant post-COVID-19 complication found in 42% of patients with severe forms of pneumonia at the 2-year evaluation. 

The severity of lung fibrosis registered a significant overall decrease at the 2-year evaluation, compared to the 3-month evaluation, with a complete regression of fibrosis observed in 37.5% of cases, most pronounced in patients in Group 2 (52.4%).

The CT quantification of pulmonary fibrosis, in conjunction with laboratory marker levels, could represent a viable strategy for predicting the progression and reversibility of the fibrotic changes and improving the monitoring of post-COVID-19 respiratory complications.

We consider that the amount of interstitial pulmonary involvement represents the optimal parameter to estimate the risk of lung fibrosis following SARS-CoV-2 pneumonia.

Follow-up evaluations of fibrosis should extend beyond two years to assess whether further regression is possible.

## Figures and Tables

**Table 1:** Technical parameters of CT scans.

Parameters	CT Scan Values
Slice thickness (mm)	3
Reconstruction thickness (mm)	1.5
Collimation	1.2
Reference mAs	250
Reference kV	120
Rotation time (s)	0.5
Pitch	0.35
FOV	Encompassed on both lungs
Reconstruction kernel	H31f for mediastinum and H60f for lung

Abbreviations: mAs—milliangstroms; kV—kilovolts; FOV—field of view.

**Table 2:** Biological parameters in the study groups.

Parameter	Admission Values (All Patients) Median [Q1; Q3]	Group 1 Median [Q1; Q3]	Group 2 Median [Q1; Q3]	Group 3 Median [Q1; Q3]
Hospital stay (days)	11 [7; 19]	12 [8; 20]	11 [6; 16]	12 [7; 19]
Duration of antiviral treatment (days)	4 [3; 6]	5 [4; 6]	4 [3; 5]	4 [2; 6]
Duration of corticotherapy (days)	7 [1; 12]	7 [1; 13]	7 [3; 11]	7 [1; 12]
C reactive protein (mg/L)	39.7 [11.5; 80.1]	40.7 [12.1; 76.7]	38.5 [8.7; 84]	42.9 [13; 83.1]
Erythrocyte Sedimentation Ratio (mm/h)	41.5 [17.9; 56]	40 [19; 57.5]	40.5 [16; 52]	44 [18.5; 56.5]
Fibrinogen (mg/dL)	454 [340; 576]	474 [359.5; 588.5]	422 [317; 531]	476 [362; 607.7]
Lactate dehydrogenase (U/L)	263 [218; 343.7]	271 [227; 355]	243 [203; 306]	281 [225; 372]
Aspartat aminotransferase (U/L)	39 [30; 54]	42 [33; 56]	36 [28; 48]	41.5 [32; 58]
Alanine aminotransferase (U/L)	38 [25; 54]	39.5 [26; 56.2]	35.5 [22.2; 46]	42 [26.2; 56]
Creatine kinase (U/L)	104 [47; 155.2]	105 [47; 161]	96.5 [45.5; 140.7]	108.5 [47.5; 165.7]
Erythrocytes (×10^6^/µL)	4.4 [3.9; 4.9]	4.5 [4.1; 4.9]	4.66 [4.2; 5.1]	4.1 [3.7; 4.6]
Leukocytes (×10^3^/µL)	6.5 [5.2; 9.6]	6.8 [5.3; 9.8]	6 [4.8; 9.1]	7.2 [5.3; 10.3]
Lymphocytes (×10^3^/µL)	0.9 [0.6; 0.9]	0.9 [0.6; 1.5]	0.8 [0.5; 1.3]	1 [0.6; 1.5]
Neutrophils (×10^3^/µL)	4.6 [3.2; 7.3]	4.8 [3.3; 7.3]	4.4 [3; 6.6]	4.8 [3.5; 7.7]
Platelets (×10^3^/µL)	189 [140; 237.7]	196.5 [145; 250.6]	200 [148.2; 255.2]	176.5 [125; 212]
Serum ferritin (ng/mL)	627.5 [305.2; 1126.3]	643 [316; 1173.5]	575 [277.7; 1092]	607 [308; 1146.7]
D-dimers (ng/mL)	193 [143.7; 264.5]	194 [144.5; 275.5]	181 [137.5; 236.5]	205.5 [145.5; 285.5]
IL-1 (pg/mL)	3.2 [0.3; 13.1]	2.6 [0.2; 13.8]	2.1 [0.3; 12.2]	4.7 [0.7; 15.3]
IL-6 (pg/mL)	65.5 [32; 218.3]	67.3 [32; 237]	65.8 [31; 209.5]	67.9 [33.5; 220.1]

Abbreviations: IL—interleukin; Q1—first quartile; Q3—third quartile.

**Table 3:** Statistical variation of the clinical and biologic parameters.

Parameter	All Patients (*p*-Value)	Group 1 vs. 2 (*p*-Value)	Group 2 vs. 3 (*p*-Value)	Group 1 vs. 3 (*p*-Value)
Hospital stay (days)	0.063	0.029	0.066	0.709
Duration of antiviral treatment (days)	0.350	0.657	0.356	0.156
Duration of corticotherapy (days)	0.998	0.898	0.995	0.890
C reactive protein (mg/L)	0.767	0.666	0.469	0.759
Erythrocyte Sedimentation Ratio (mm/h)	0.234	0.174	0.117	0.803
Fibrinogen (mg/dL)	0.008	0.019	0.003	0.522
Lactate dehydrogenase (U/L)	0.013	0.016	0.007	0.780
Aspartat aminotransferase (U/L)	0.030	0.033	0.016	0.829
Alanine aminotransferase (U/L)	0.033	0.042	0.015	0.686
Creatine kinase (U/L)	0.847	0.643	0.597	0.949
Erythrocytes (×10^6^/µL)	<0.001	0.100	<0.001	<0.001
Leukocytes (×10^3^/µL)	0.410	0.269	0.230	0.924
Lymphocytes (×10^3^/µL)	0.459	0.275	0.286	0.979
Neutrophils (×10^3^/µL)	0.417	0.234	0.273	0.927
Platelets (×10^3^/µL)	0.037	0.713	0.017	0.044
Serum ferritin (ng/mL)	0.942	0.792	0.950	0.743
D-dimers (ng/mL)	0.220	0.164	0.111	0.845
IL-1 (pg/mL)	0.730	0.672	0.430	0.749
IL-6 (pg/mL)	0.999	0.973	0.990	0.964

Abbreviations: IL—interleukin.

**Table 4:** Radiological parameters in the study groups.

Parameter	Admission Values (All Patients) Median [Q1; Q3]	Group 1 Median [Q1; Q3]	Group 2 Median [Q1; Q3]	Group 3 Median [Q1; Q3]
Pulmonary lobes with pneumonia (*n*, median, Q1, Q3)	5 [4; 5]	5 [4; 5]	4 [4; 5]	5 [5; 5]
Alveolar consolidation (%, median, Q1, Q3)	0.9 [0.7; 1.6]	1 [0.7; 1.7]	0.8 [0.6; 1.5]	1.1 [0.7; 1.7]
Mixed lesions (%, median, Q1, Q3)	2.5 [1.3; 3.8]	2.6 [1.5; 3.9]	2.2 [1.1; 3.2]	2.6 [1.5; 4.1]
Interstitial involvement (%, median, Q1, Q3)	31.5 [22; 43.9]	32.3 [23.3; 45.8]	27.6 [19.8; 39.6]	34.3 [22.6; 47.3]
Normal lung densities (%, median, Q1, Q3)	59.6 [45.4; 68.7]	58.7 [44.3; 67.6]	63.9 [51.2; 71.4]	56.2 [41.4; 68.5]
Total lung involvement (%, median, Q1, Q3)	35.5 [24.6; 50.5]	36.2 [26.3; 53.2]	31.4 [22.7; 44.9]	38.4 [25.2; 52.8]

Abbreviations: Q1—first quartile; Q3—third quartile.

**Table 5:** Statistical variation of the radiological parameters.

Parameter	All Patients (*p*-Value)	Group 1 vs. 2 (*p*-Value)	Group 2 vs. 3 (*p*-Value)	Group 1 vs. 3 (*p*-Value)
Pulmonary lobes with pneumonia	<0.001	<0.001	<0.001	0.598
Alveolar consolidation	0.271	0.148	0.179	0.918
Mixed lesions	0.209	0.136	0.116	0.934
Interstitial involvement	<0.001	0.001	<0.001	0.594
Normal lung densities	<0.001	0.001	<0.001	0.596
Total pulmonary involvement	0.001	0.004	0.001	0.641

**Table 6:** Lung fibrosis in the study groups.

Parameter	All Patients		Group 1		Group 2		Group 3	
	3 Months	2 Years	3 Months	2 Years	3 Months	2 Years	3 Months	2 Years
No fibrosis	135 (32.1%)	242 (57.6%)	42 (30%)	75 (53.6%)	58 (41.4%)	101 (72.1%)	35 (25%)	66 (47.1%)
Mild fibrosis (*n*,%) (1–9%)	147 (35%)	173 (41.2%)	43 (30.7%)	63 (45%)	51 (36.4%)	39 (27.8%)	53 (37.8%)	71 (50.7%)
Moderate fibrosis (*n*,%) (9–20%)	89 (21.2%)	5 (1.2%)	34 (24.3%)	2 (1.4%)	23 (16.4)	0 (0%)	32 (22.8%)	3 (2.1)
Severe fibrosis (*n*,%) (over 20%)	49 (11.6%)	0 (0%)	21 (15%)	0 (0%)	8 (5.7%)	0 (0%)	20 (14.3)	0 (0%)
Ground-glass pattern	260 (61.9%)	116 (27.6)	89 (63.6%)	32 (22.8%)	71 (50.7%)	33 (23.6%)	100 (71.4%)	51 (36.4)
Linear/reticular pattern	246 (58.5%)	167 (39.7)	92 (65.7%)	61 (43.6%)	78 (55.7%)	39 (27.8%)	76 (54.3%)	67 (47.8)
Honeycomb pattern	16 (3.8%)	16 (3.8%)	6 (4.3%)	6 (4.3%)	3 (2.1%)	3 (2.1%)	7 (5%)	7 (5%)
Bronchiectasis	138 (32.8%)	66 (15.7%)	51 (36.4%)	18 (12.8%)	42 (30%)	20 (14.3)	45 (32.1%)	28 (20%)

**Table 7:** Risk factors associated with pulmonary fibrosis at the 3-month and 2-year follow-ups.

Parameter	3-Month Fibrosis			2-Year Fibrosis		
	Spearman’s Rho	*p*-Value	OR [CI]	Spearman’s Rho	*p*-Value	OR [CI]
Age	0.261	<0.001	1034 [1017; 1051]	0.159	0.001	1021 [1006; 1036]
Hospital stay	0.447	<0.001	1053 [1020; 1088]	0.241	<0.001	1048 [1021; 1076]
CRP	0.267	<0.001	1018 [1011; 1024]	0.081	0.113	1005 [1002; 1009]
ESR	0.451	<0.001	1051 [1035; 1066]	0.233	<0.001	1022 [1011; 1033]
Fibrinogen	0.273	<0.001	1004 [1002; 1005]	0.171	<0.001	1002 [1001; 1004]
LDH	0.392	<0.001	1008 [1006; 1011]	0.248	<0.001	1003 [1002; 1005]
Aspartat aminotransferase	0.138	0.005	1010 [1001; 1020]	0.144	0.004	1009 [1000; 1018]
Alanine aminotransferase	0.171	<0.001	1012 [1004; 1021]	0.104	0.033	1006 [0.998; 1013]
Creatine kinase	−0.058	0.237	0.999 [0.998; 1000]	0.033	0.501	0.999 [0.998; 1000]
Erythrocytes	−0.014	0.782	0.994 [0.712; 1388]	−0.047	0.341	0.877 [0.639; 1205]
Leukocytes	0.269	<0.001	1044 [0.993; 1097]	0.093	0.058	1029 [0.986; 1074]
Lymphocytes	0.097	0.046	1649 [1174; 2315]	0.037	0.455	1382 [1036; 1845]
Neutrophils	0.234	<0.001	1030 [0976; 1086]	0.098	0.046	1028 [0.980; 1078]
Platelets	0.047	0.338	1001 [1000; 1003]	−0.014	0.769	1000 [0.998; 1002]
Serum ferritin	0.052	0.346	1000 [1000; 1001]	0.049	0.380	1000 [1000; 1001]
D-dimers	0.277	<0.001	1004 [1002; 1007]	0.203	<0.001	1004 [1002; 1006]
IL-1	−0.125	0.141	0.999 [0.990; 1009]	0.018	0.829	0.999 [0.989; 1008]
IL-6	0.188	0.002	1000 [0.999; 1000]	0.067	0.269	1000 [1000; 1001]
Duration of antiviral treatment	0.273	<0.001	1075 [1002; 1154]	0.260	<0.001	1116 [1047; 1189]
Duration of corticotherapy	0.302	<0.001	1040 [1005; 1077]	0.065	0.210	1009 [0.979; 1040]
Pulmonary lobes with pneumonia	0.393	<0.001	1873 [1521; 2307]	0.334	<0.001	2377 [1780; 3175]
Alveolar consolidation	0.321	<0.001	1063 [0.893; 1266]	0.272	<0.001	1375 [1152; 1642]
Mixed pulmonary lesions	0.448	<0.001	1104 [1011; 1206]	0.350	<0.001	1207 [1111; 1311]
Interstitial pulmonary involvement	0.732	<0.001	1130 [1100; 1160]	0.475	<0.001	1072 [1054; 1092]
Total pulmonary involvement	0.696	<0.001	1089 [1068; 1111]	0.476	<0.001	1060 [1045; 1076]

Abbreviations: CI—confidence interval; CRP—C reactive protein, ESR—erythrocyte sedimentation rate; IL—interleukin; LDH—lactate dehydrogenase; OR—odds ratio.

**Table 8:** ROC curve performance in evaluating fibrosis for registered parameters.

Parameter	3 Months Fibrosis			2 Years Fibrosis		
	AUC	CI 95%	*p*-Value	AUC	CI 95%	*p*-Value
Age	0.629	[0.573; 0.685]	<0.001	0.579	[0.523; 0.653]	0.006
Hospital stay	0.633	[0.564; 0.703]	<0.001	0.597	[0.536; 0.657]	0.002
ESR	0.769	[0.709; 0.829]	<0.001	0.622	[0.560; 0.685]	<0.001
Fibrinogen	0.655	[0.600; 0.710]	<0.001	0.600	[0.545; 0.654]	<0.001
LDH	0.694	[0.641; 0.747]	<0.001	0.634	[0.580; 0.689]	<0.001
Aspartat transaminase	0.604	[0.541; 0.667]	0.001	0.586	[0.531; 0.641]	0.003
Alanine transaminase	0.628	[0.567; 0.689]	<0.001	0.548	[0.493; 0.604]	0.092
Lymphocytes	0.565	[0.508; 0.621]	0.031	0.535	[0.479; 0.592]	0.214
Neutrophils	0.573	[0.515; 0.631]	0.016	0.547	[0.490; 0.604]	0.102
D-dimers	0.613	[0.556; 0.670]	<0.001	0.624	[0.570; 0.679]	<0.001
Duration of antiviral treatment	0.625	[0.558; 0.691]	<0.001	0.622	[0.564; 0.681]	<0.001
Pulmonary lobes with pneumonia	0.664	[0.607; 0.722]	<0.001	0.663	[0.612; 0.714]	<0.001
Alveolar consolidation	0.639	[0.579; 0.698]	<0.001	0.630	[0.574; 0.685]	<0.001
Mixed pulmonary lesions	0.700	[0.639; 0.761]	<0.001	0.676	[0.624; 0.728]	<0.001
Interstitial pulmonary involvement	0.831	[0.788; 0.873]	<0.001	0.728	[0.679; 0.777]	<0.001
Total pulmonary involvement	0.808	[0.760; 0.857]	<0.001	0.731	[0.682; 0.780]	<0.001

Abbreviations: AUC—area under the curve; CI—confidence interval; ESR—erythrocyte sedimentation rate; LDH—lactate dehydrogenase.

**Table 9:** Multivariable logistic regression model for lung fibrosis at the 3-month evaluation.

Variable	B	S.E.	Wald	*p*	OR	95% CI for OR	
						Lower	Upper
CRP	0.026	0.007	13,994	<0.001	1026	1012	1040
LDH	0.009	0.003	9354	0.002	1009	1003	1014
AST	–0.031	0.009	11,907	0.001	0.970	0.953	0.987
Lymphocytes	1670	0.551	9194	0.002	5313	1805	15,639
Interstitial pulmonary involvement	0.084	0.020	17,960	<0.001	1087	1046	1130
Hospital stay	0.048	0.035	1965	0.161	1050	0.981	1123
Constant	−5819	1082	28,933	<0.001	0.003		

Abbreviations: AST—aspartate aminotransferase; CI—confidence interval; CRP—C reactive protein; LDH—lactate dehydrogenase; OR—odds ratio.

**Table 10:** Multivariable logistic regression model for lung fibrosis at the 2-year evaluation.

Variable	B	S.E.	Wald	*p*	OR	95% CI for OR	
						Lower	Upper
Lymphocytes	0.412	0.185	4928	0.026	1509	1049	2171
Duration of corticotherapy	−0.059	0.022	7058	0.008	0.943	0.903	0.985
Pulmonary lobes with pneumonia	0.612	0.177	12,005	0.001	1844	1305	2607
Interstitial pulmonary involvement	0.062	0.011	30,754	<0.001	1064	1041	1088
Constant	−6890	1870	13,578	<0.001	0.001		

Abbreviations: CI—confidence interval; OR—odds ratio.

## Data Availability

The data presented in this article are available upon request from the corresponding author.

## Supplementary Materials

The following supporting information can be downloaded at: https://www.mdpi.com/article/10.3390/diagnostics14242811/s1, Figure S1: ROC curve—interstitial involvement—3months fibrosis; Figure S2: ROC curve—interstitial involvement—2 years fibrosis; Figure S3: ROC curve—age—3months fibrosis; Figure S4: ROC curve—hospital stay—3months fibrosis; Figure S5: ROC curve—ESR—3months fibrosis; Figure S6: ROC curve—fibrinogen—3months fibrosis; Figure S7: ROC curve—LDH—3months fibrosis; Figure S8: ROC curve—aspartat transaminase—3months fibrosis; Figure S9: ROC curve—alanine transaminase—3months fibrosis; Figure S10: ROC curve—lymphocytes—3months fibrosis; Figure S11: ROC curve—neutrophils—3months fibrosis; Figure S12: ROC curve—D-dimers—3months fibrosis; Figure S13: ROC curve—duration of antiviral treatment—3months fibrosis; Figure S14: ROC curve—pulmonary lobes with pneumonia—3months fibrosis; Figure S15: ROC curve—alveolar consolidation—3months fibrosis; Figure S16: ROC curve—mixed pulmonary lesions—3months fibrosis; Figure S17: ROC curve—total pulmonary involvement—3months fibrosis; Figure S18: ROC curve—age—2 years fibrosis; Figure S19: ROC curve—hospital stay—2 years fibrosis; Figure S20: ROC curve—ESR—2 years fibrosis; Figure S21: ROC curve—fibrinogen—2 years fibrosis; Figure S22: ROC curve—LDH—2 years fibrosis; Figure S23: ROC curve—aspartat transaminase—2 years fibrosis; Figure S24: ROC curve—aspartat transaminase—2 years fibrosis; Figure S25: ROC curve—lymphocytes—2 years fibrosis: Figure S26: ROC curve—neutrophils—2 years fibrosis; Figure S27: ROC curve—D-dimers—2 years fibrosis; Figure S28: ROC curve—duration of antiviral treatment—2 years fibrosis; Figure S29: ROC curve—pulmonary lobes with pneumonia—2 years fibrosis; Figure S30: ROC curve—alveolar consolidation—2 years fibrosis; Figure S31: ROC curve—mixed pulmonary lesions—2 years fibrosis; Figure S32: ROC curve—total pulmonary involvement—2 years fibrosis.
