# Supplementary figures and images for: Surviving COVID-19 and Battling Fibrosis: A Retrospective Cohort Study Across Three Pandemic Waves
Source: Diagnostics (Basel). 2024 Dec 13;14(24):2811. doi: 10.3390/diagnostics14242811 (PMC11674708; doi:10.3390/diagnostics14242811)

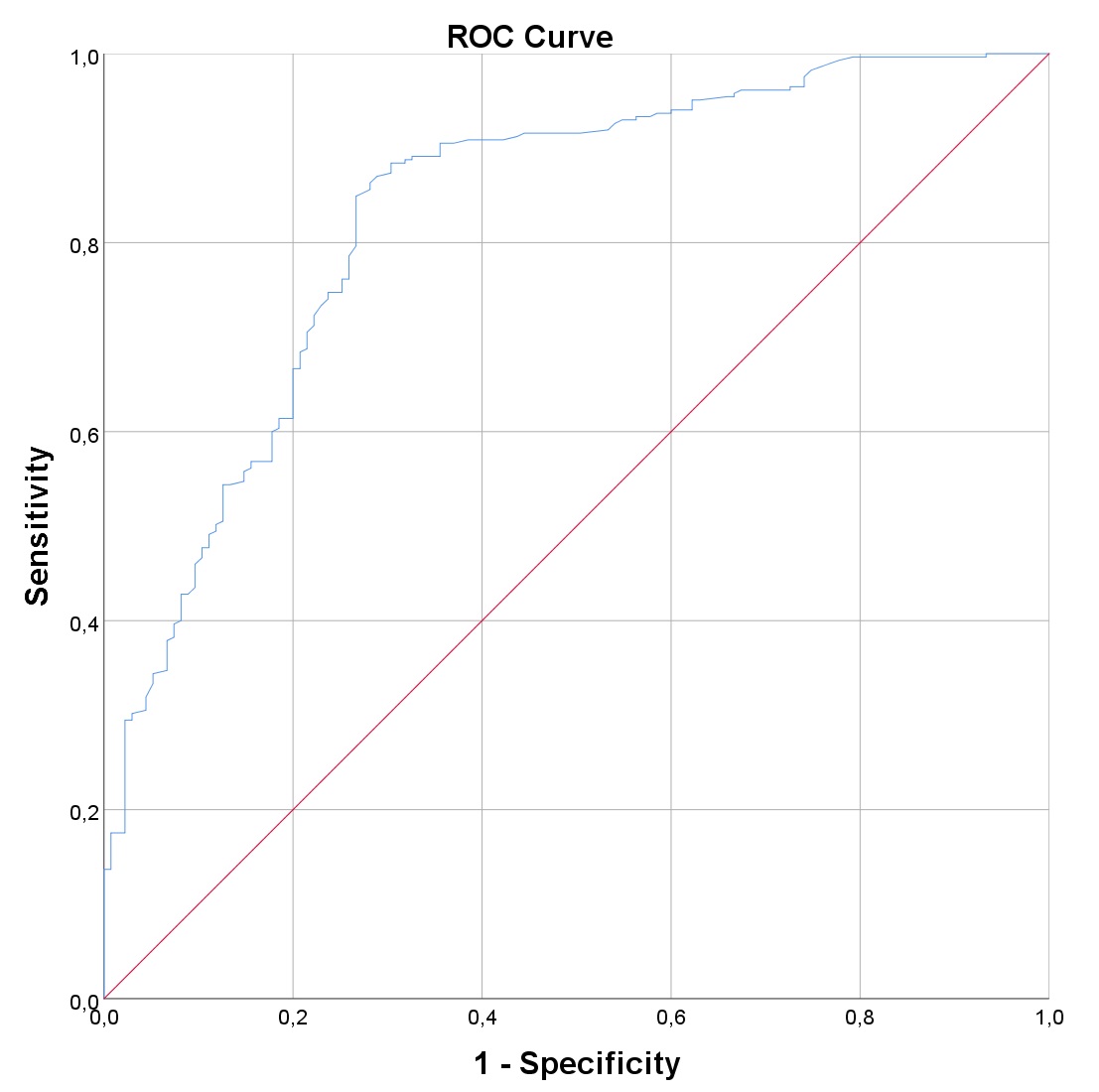

Supplement: Supplementary file 1 [file diagnostics-14-02811-s001.zip › Figure S1. ROC curve - interstitial involvement - 3months fibrosis.jpg]

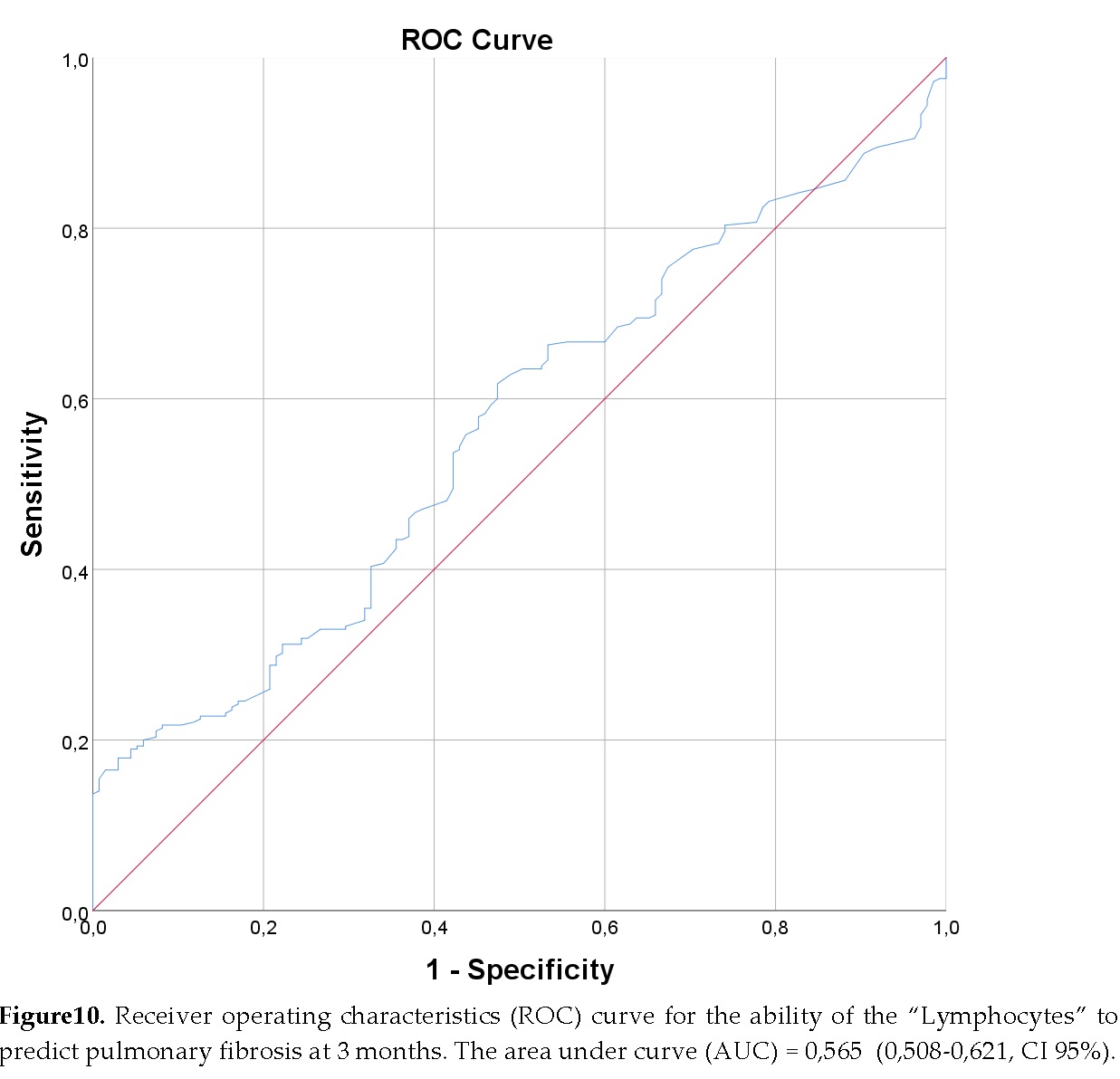

Supplement: Supplementary file 1 [file diagnostics-14-02811-s001.zip › Figure S10. ROC curve - lymphocytes - 3months fibrosis.jpg]

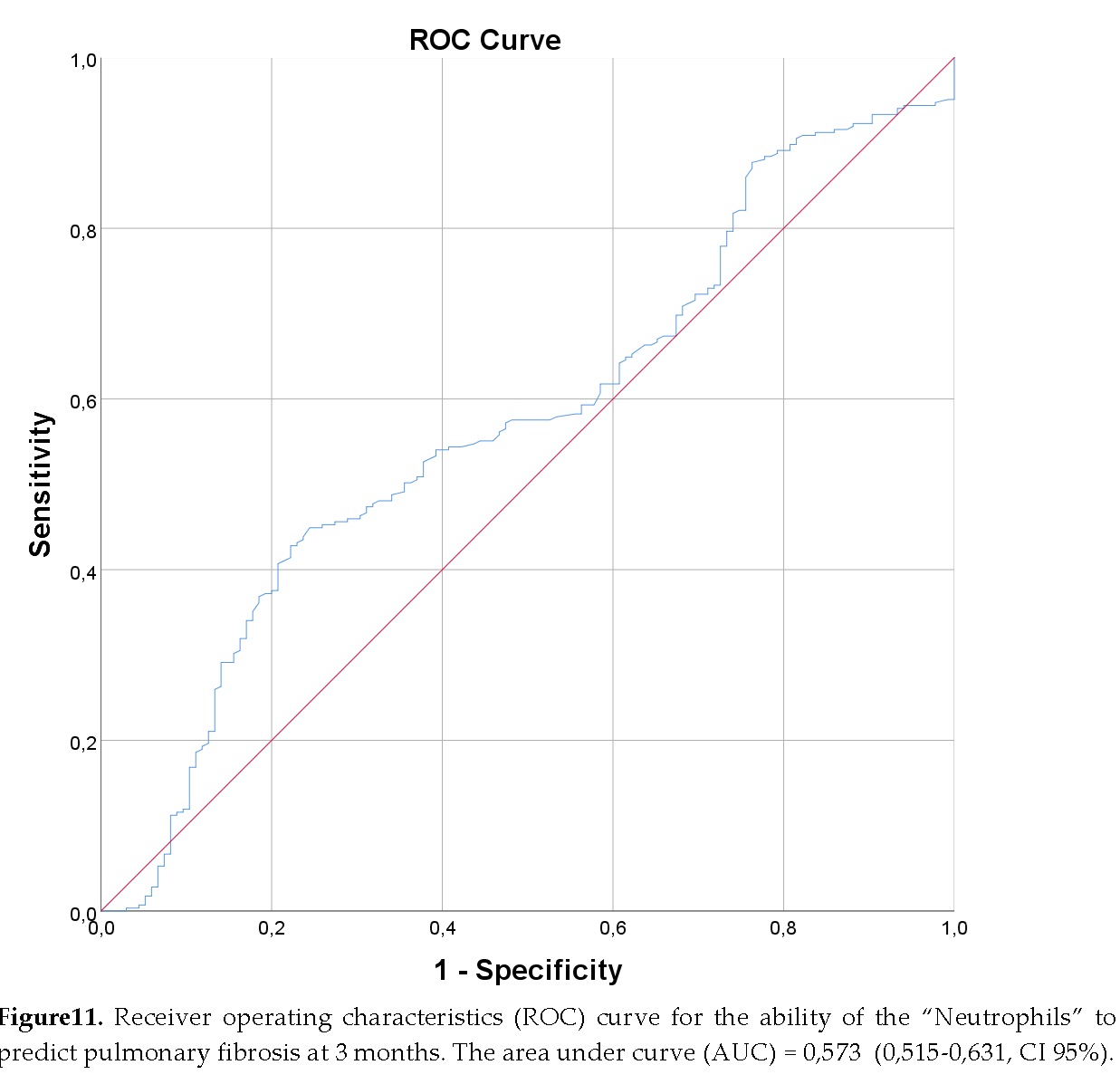

Supplement: Supplementary file 1 [file diagnostics-14-02811-s001.zip › Figure S11. ROC curve - neutrophils - 3months fibrosis.jpg]

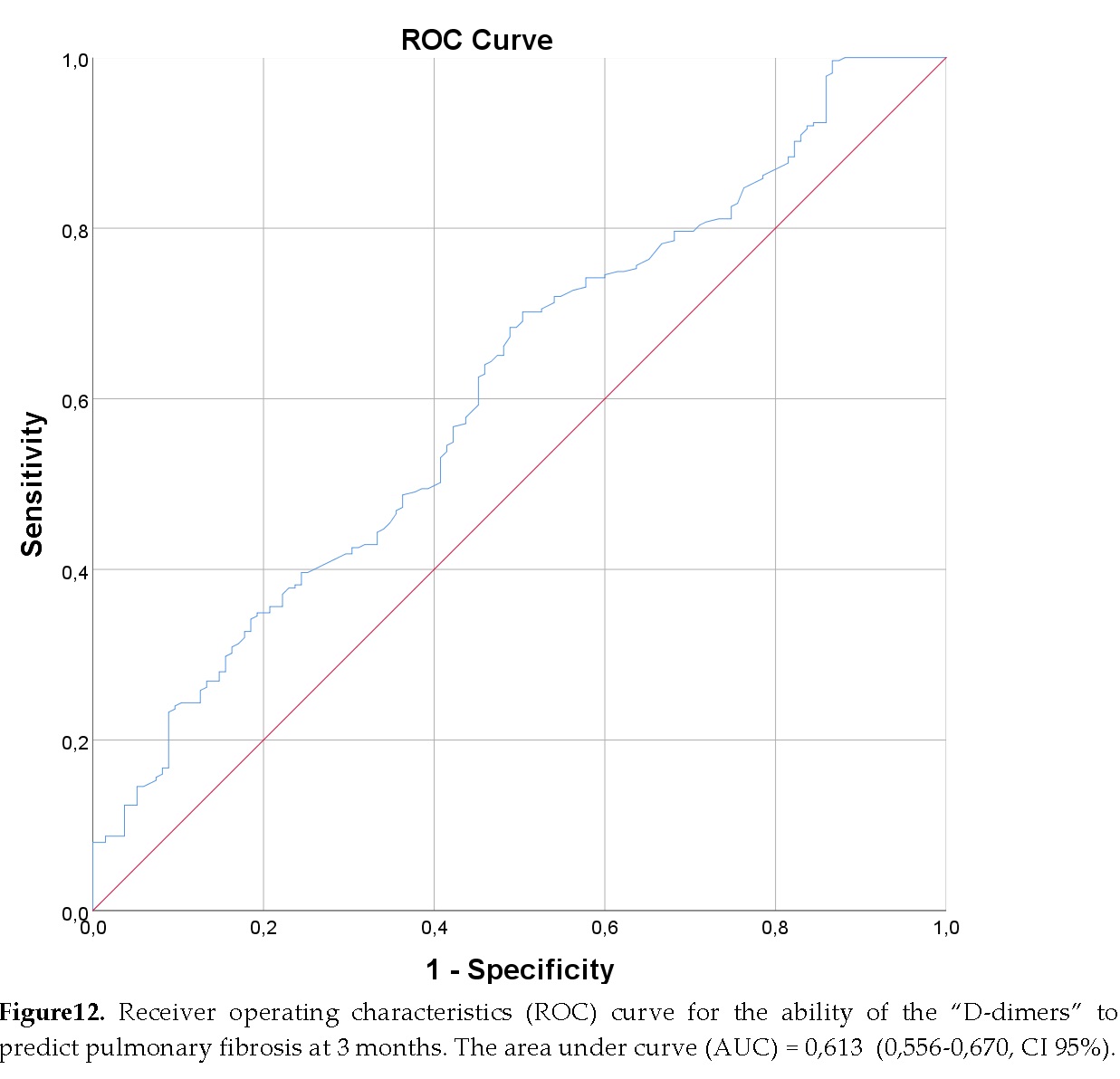

Supplement: Supplementary file 1 [file diagnostics-14-02811-s001.zip › Figure S12. ROC curve - D-dimers - 3months fibrosis.jpg]

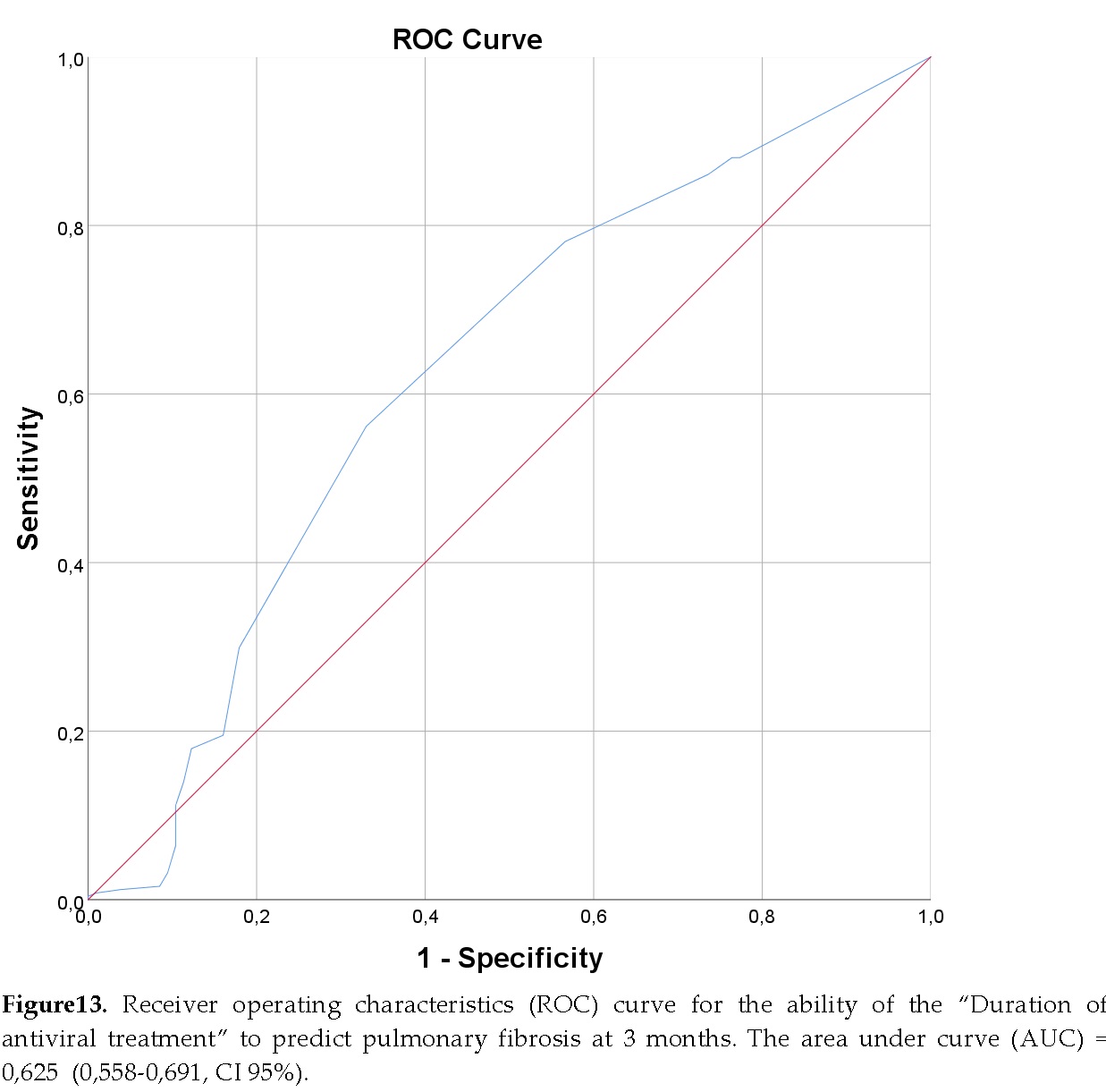

Supplement: Supplementary file 1 [file diagnostics-14-02811-s001.zip › Figure S13. ROC curve - duration of antiviral treatment - 3months fibrosis.jpg]

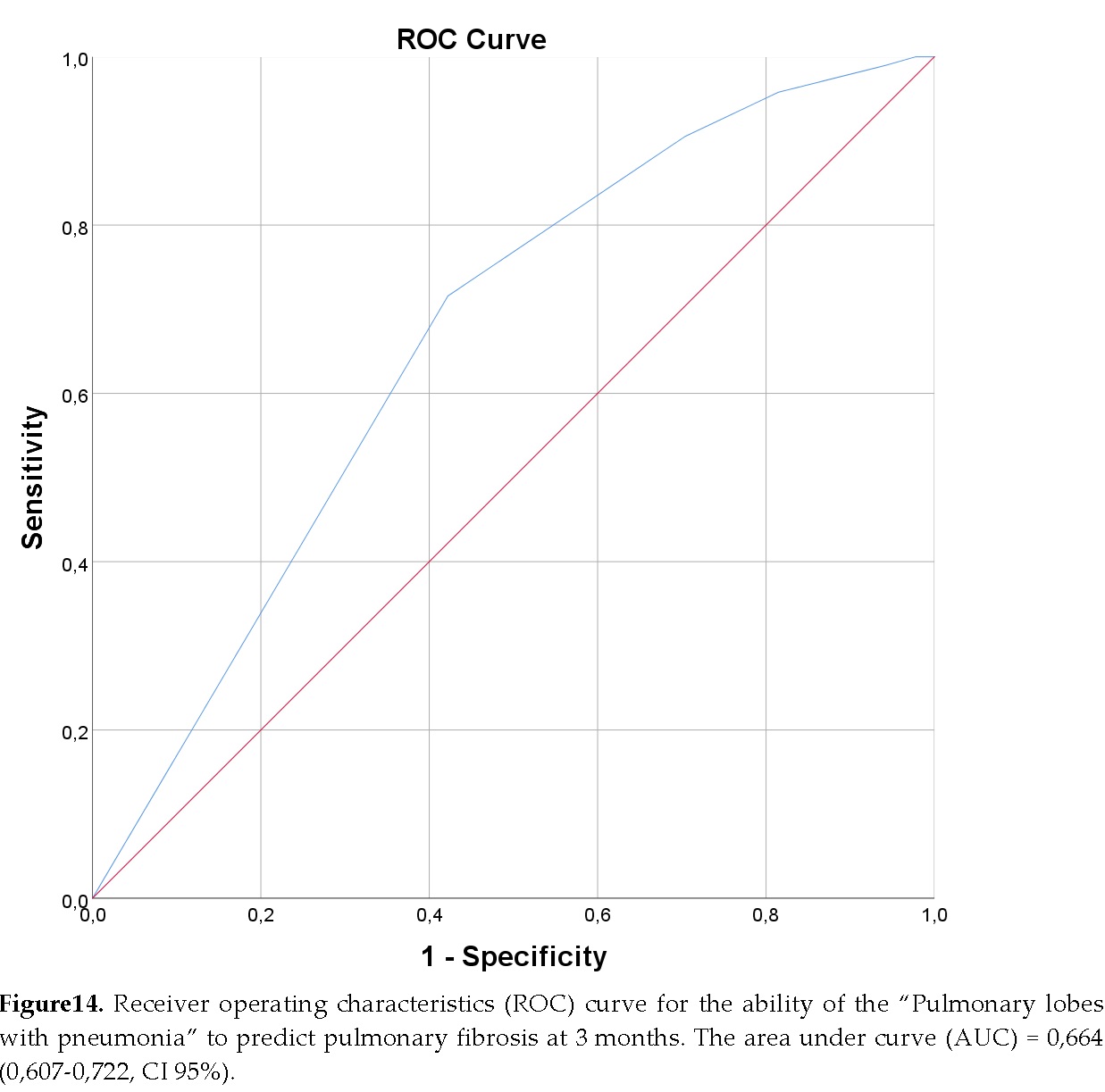

Supplement: Supplementary file 1 [file diagnostics-14-02811-s001.zip › Figure S14. ROC curve - pulmonary lobes with pneumonia - 3months fibrosis.jpg]

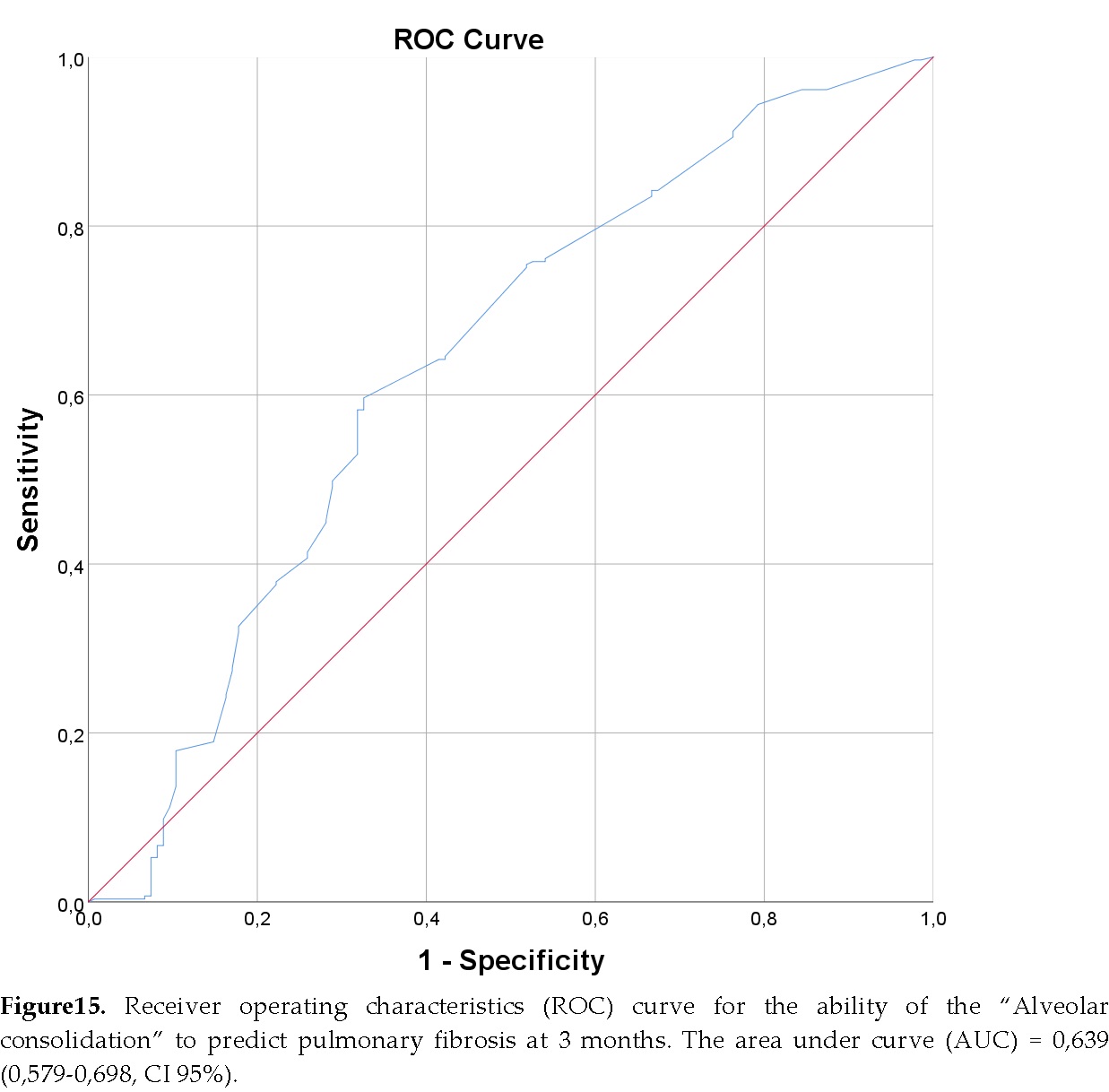

Supplement: Supplementary file 1 [file diagnostics-14-02811-s001.zip › Figure S15. ROC curve - alveolar consolidation - 3months fibrosis.jpg]

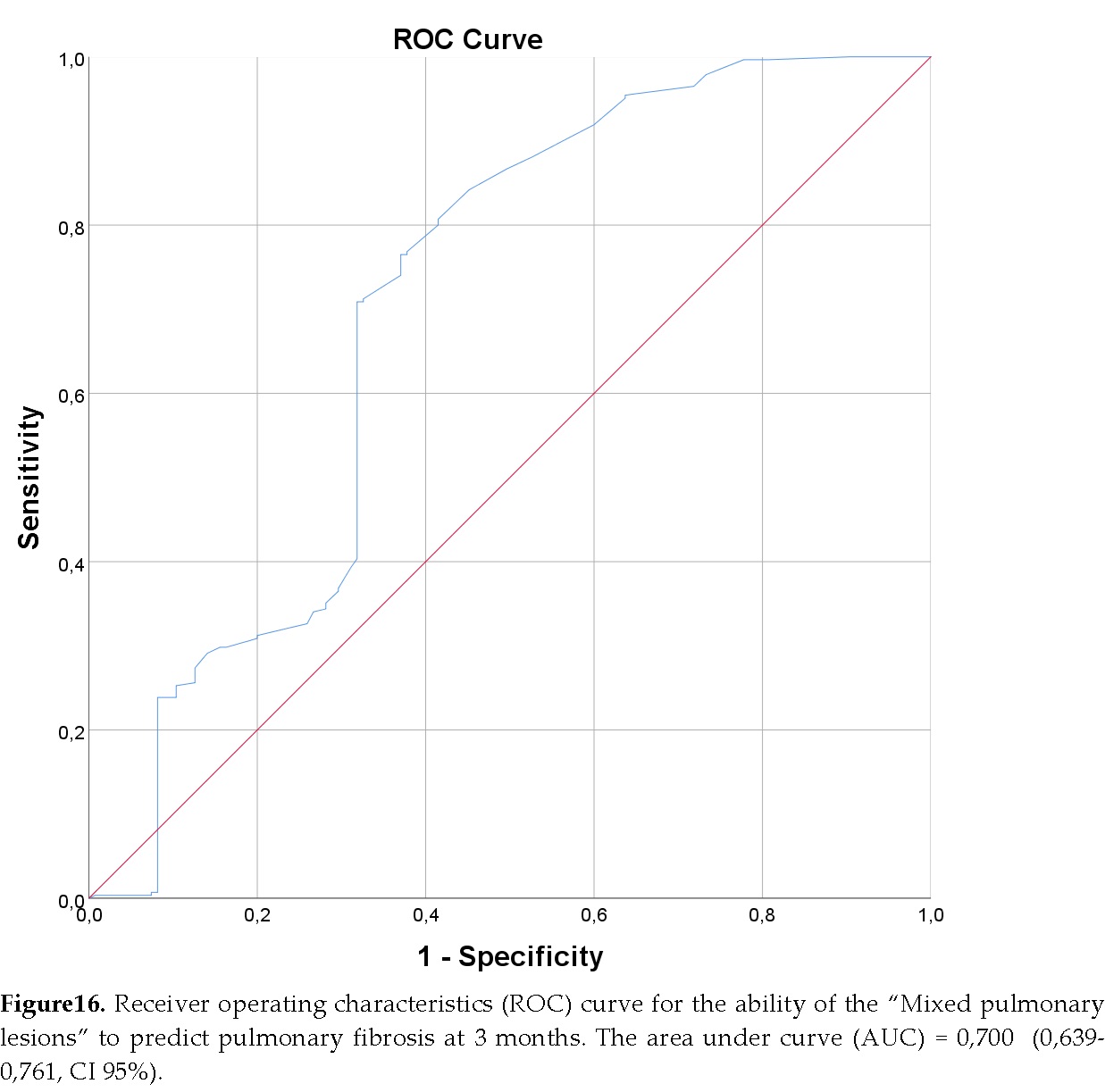

Supplement: Supplementary file 1 [file diagnostics-14-02811-s001.zip › Figure S16. ROC curve - mixed pulmonary lesions - 3months fibrosis.jpg]

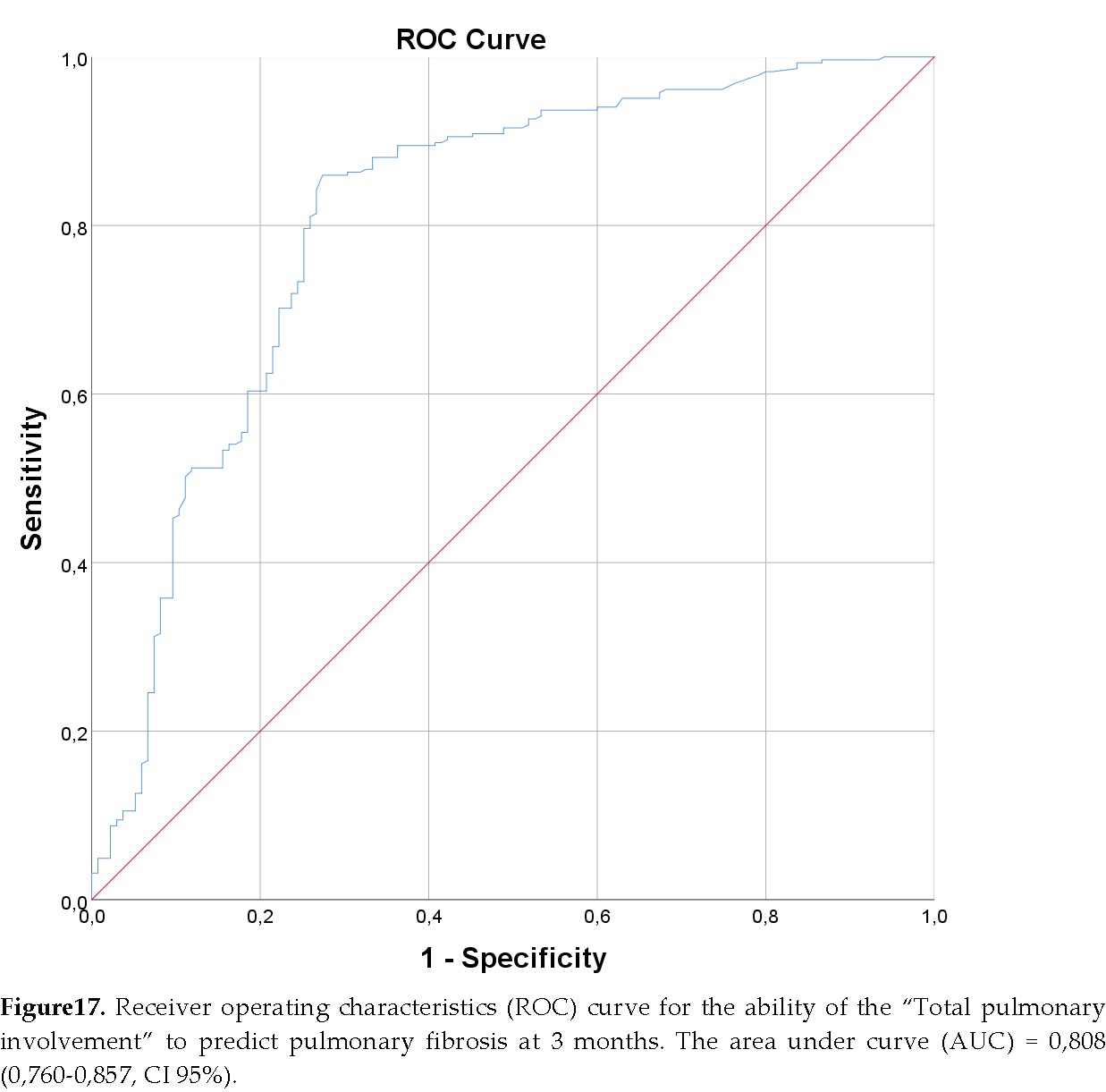

Supplement: Supplementary file 1 [file diagnostics-14-02811-s001.zip › Figure S17. ROC curve - total pulmonary involvement - 3months fibrosis.jpg]

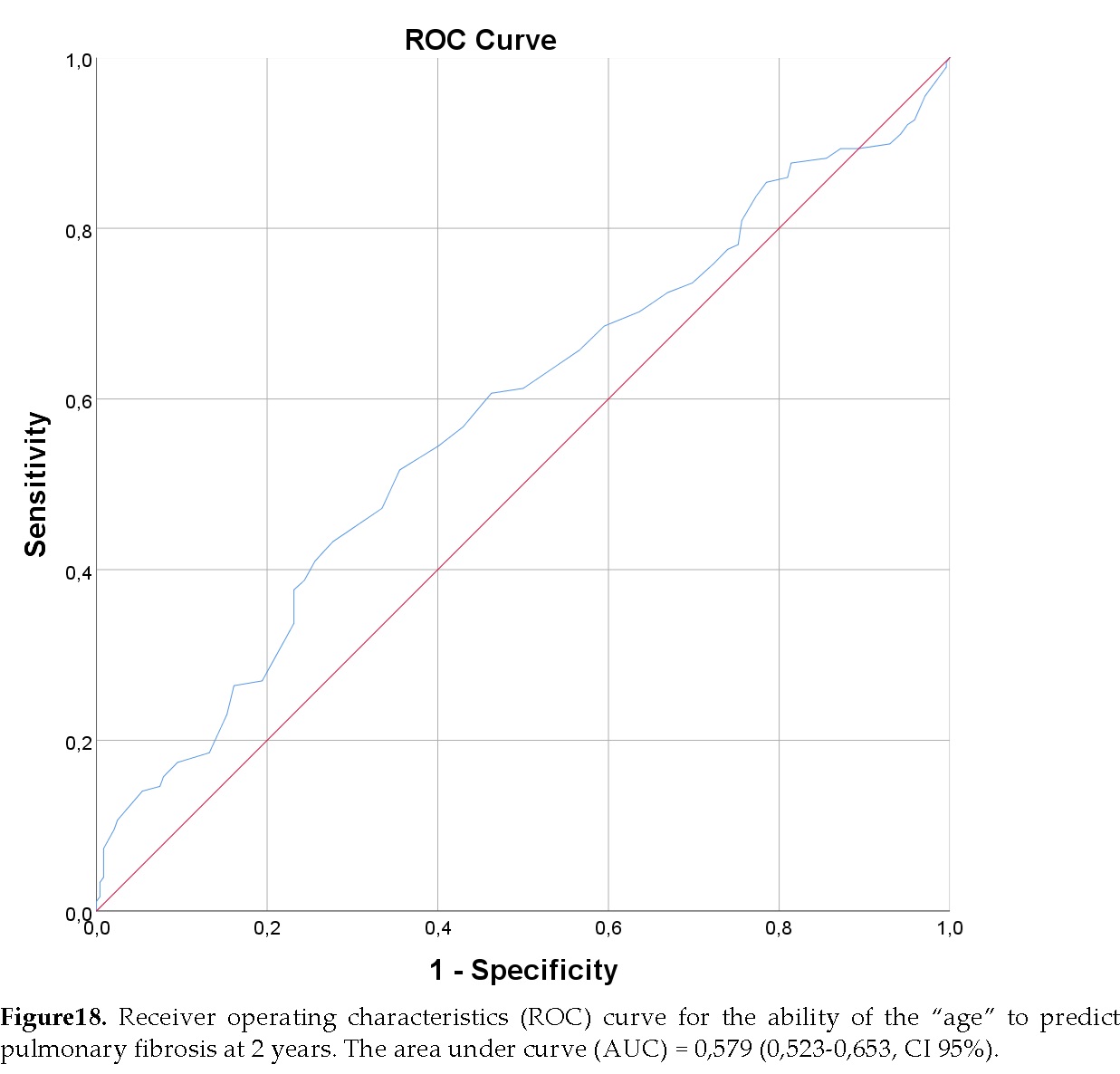

Supplement: Supplementary file 1 [file diagnostics-14-02811-s001.zip › Figure S18. ROC curve - age - 2 years fibrosis.jpg]

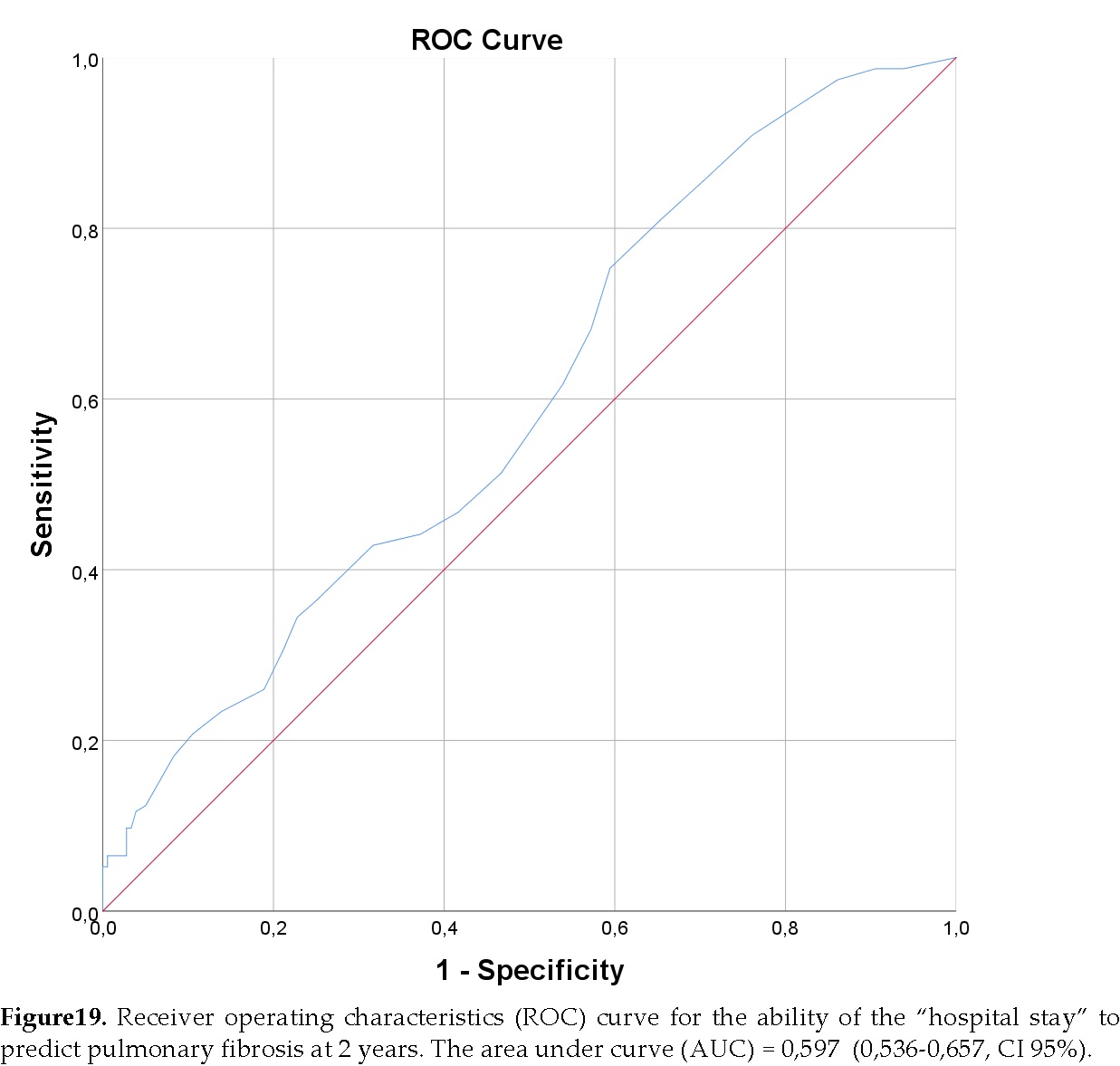

Supplement: Supplementary file 1 [file diagnostics-14-02811-s001.zip › Figure S19. ROC curve - hospital stay - 2 years fibrosis.jpg]

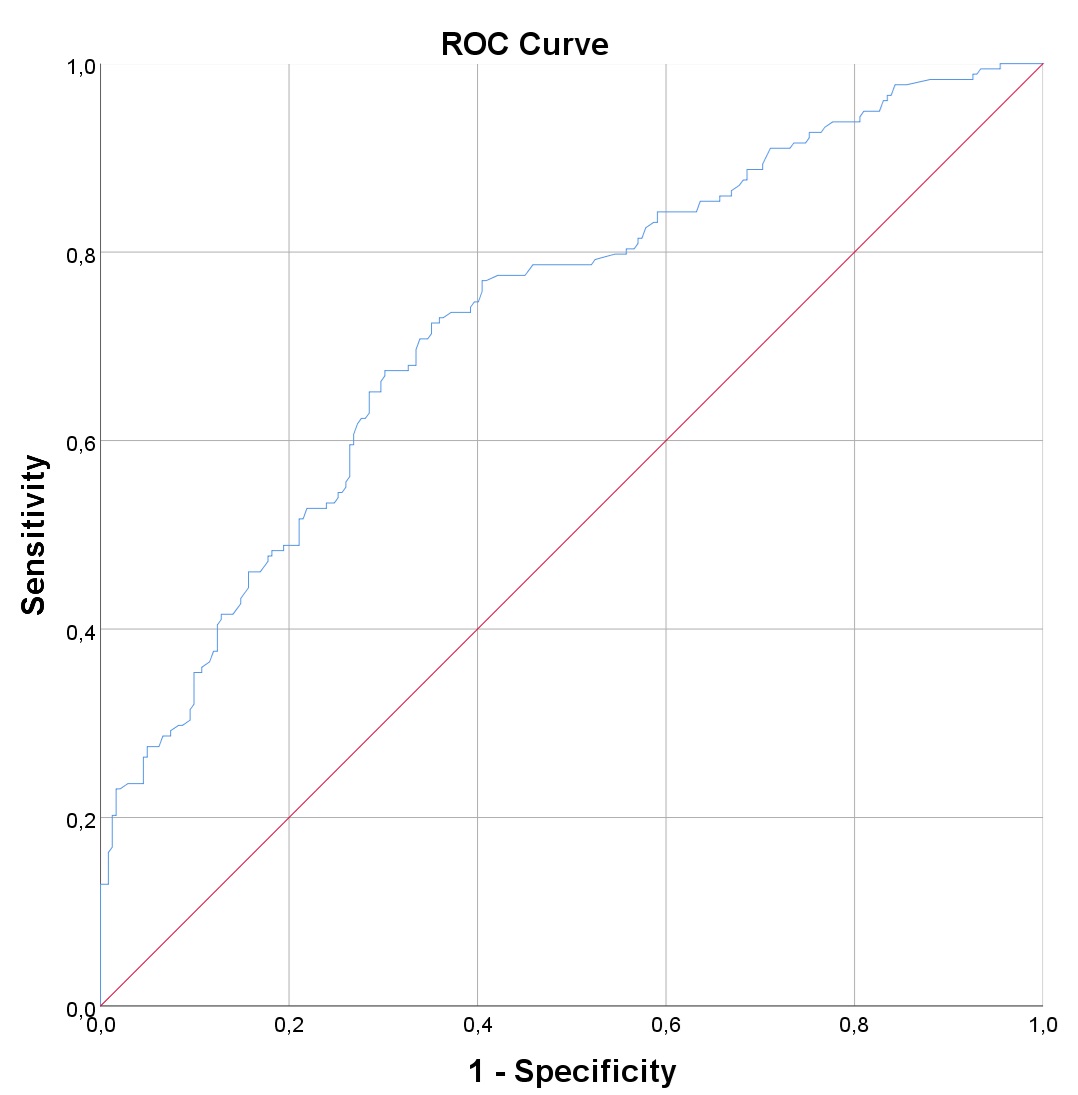

Supplement: Supplementary file 1 [file diagnostics-14-02811-s001.zip › Figure S2. ROC curve - interstitial involvement - 2 years fibrosis.jpg]

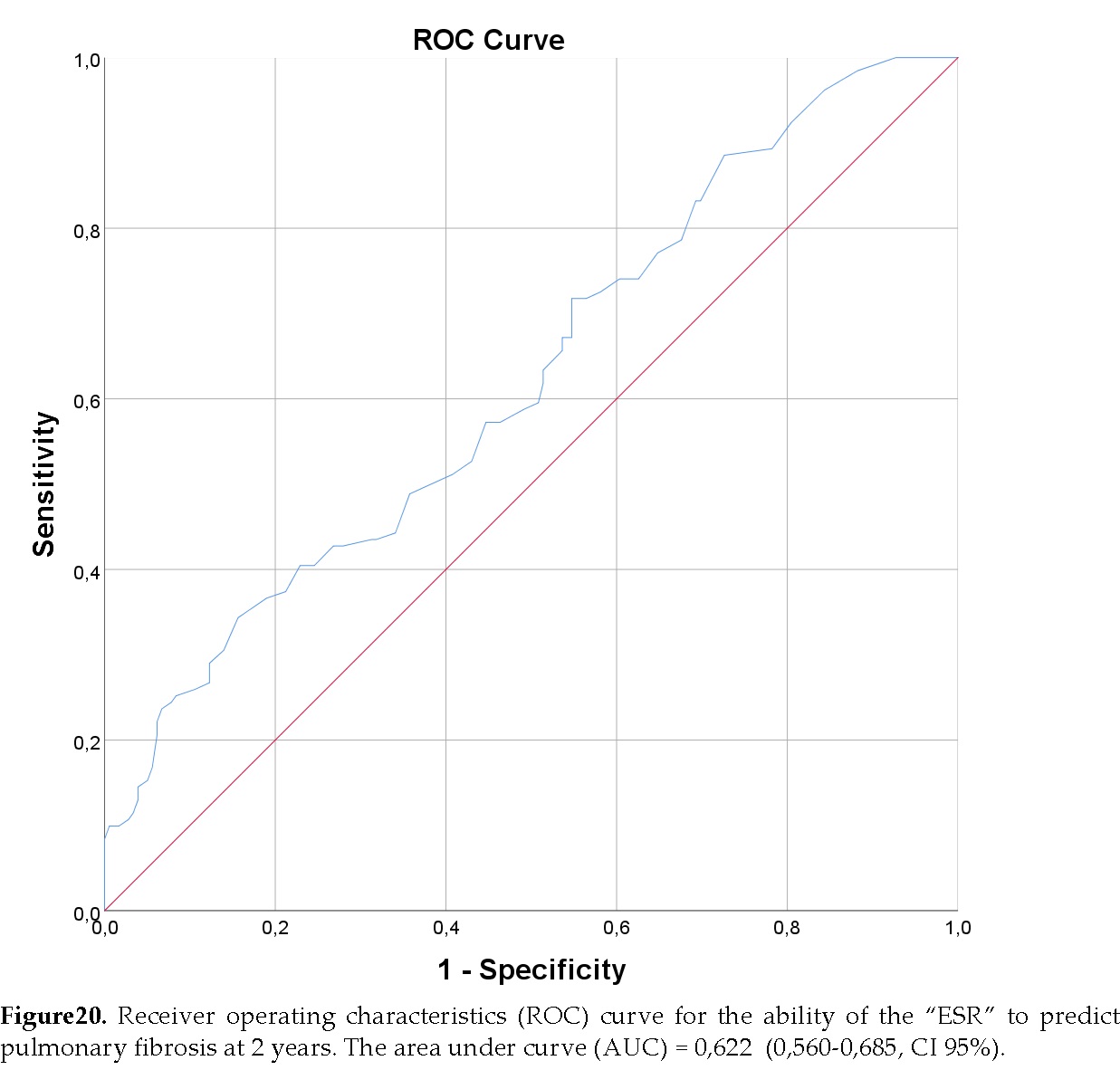

Supplement: Supplementary file 1 [file diagnostics-14-02811-s001.zip › Figure S20. ROC curve - ESR - 2 years fibrosis.jpg]

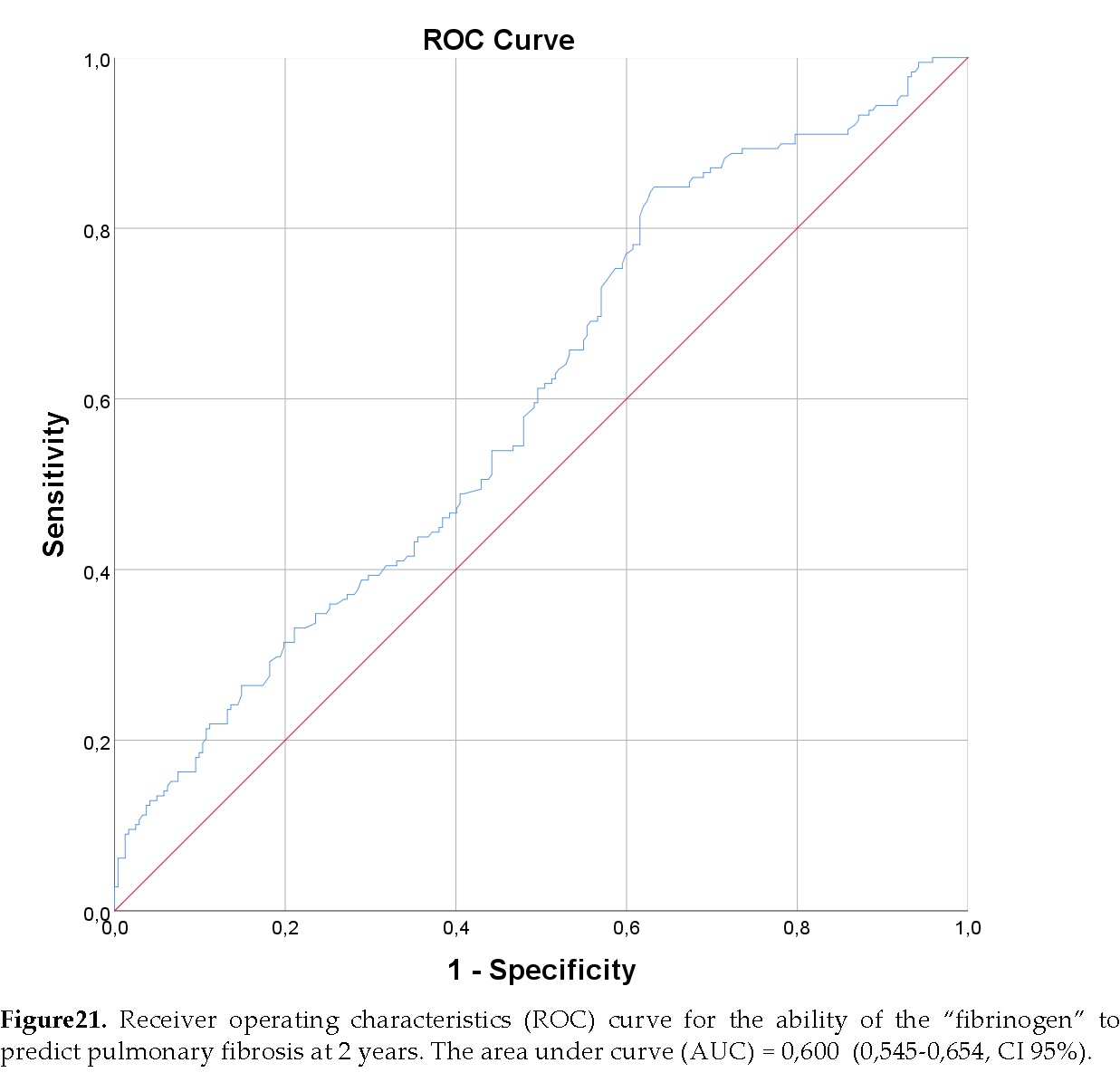

Supplement: Supplementary file 1 [file diagnostics-14-02811-s001.zip › Figure S21. ROC curve - fibrinogen - 2 years fibrosis.jpg]

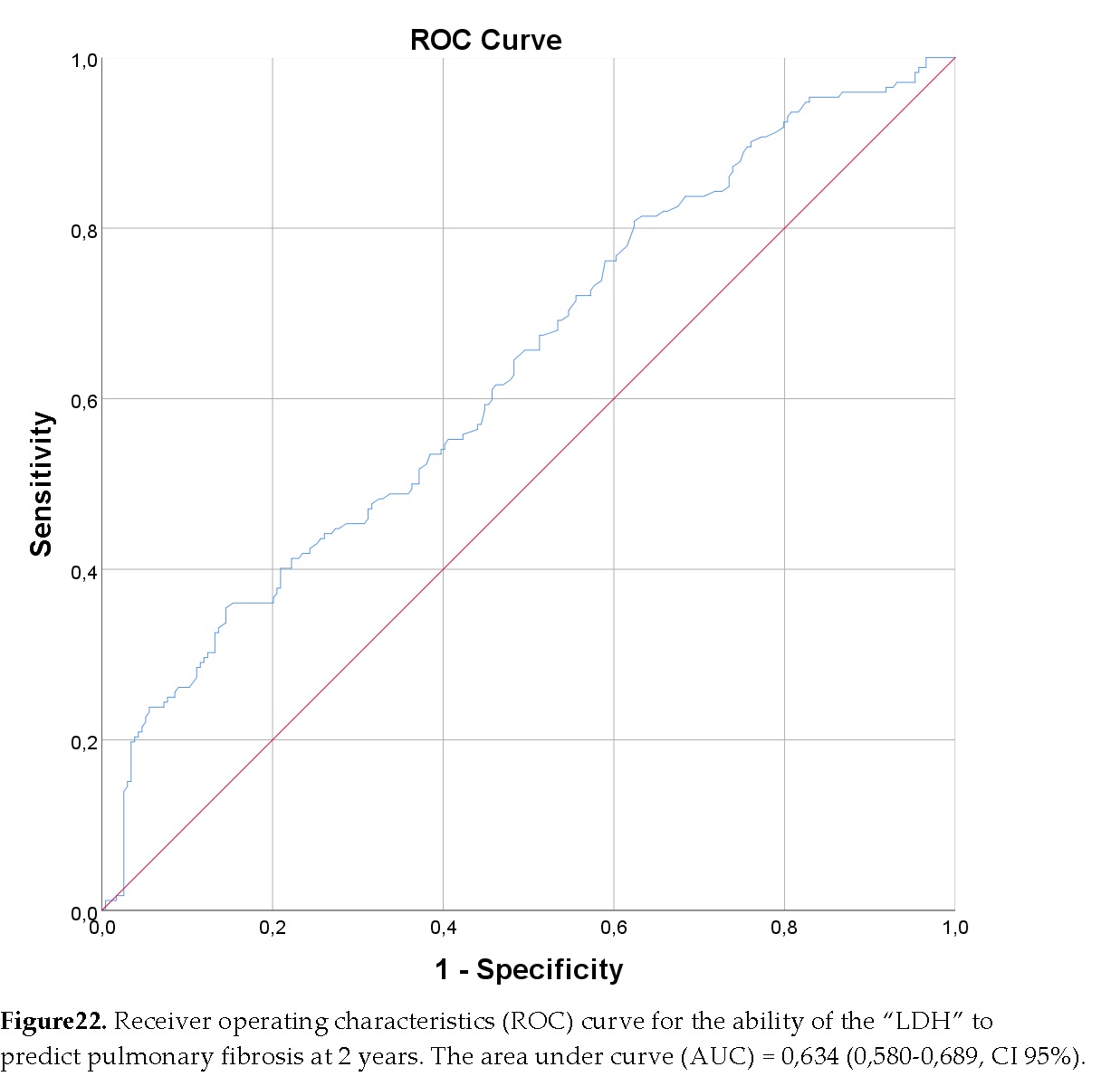

Supplement: Supplementary file 1 [file diagnostics-14-02811-s001.zip › Figure S22. ROC curve - LDH - 2 years fibrosis.jpg]

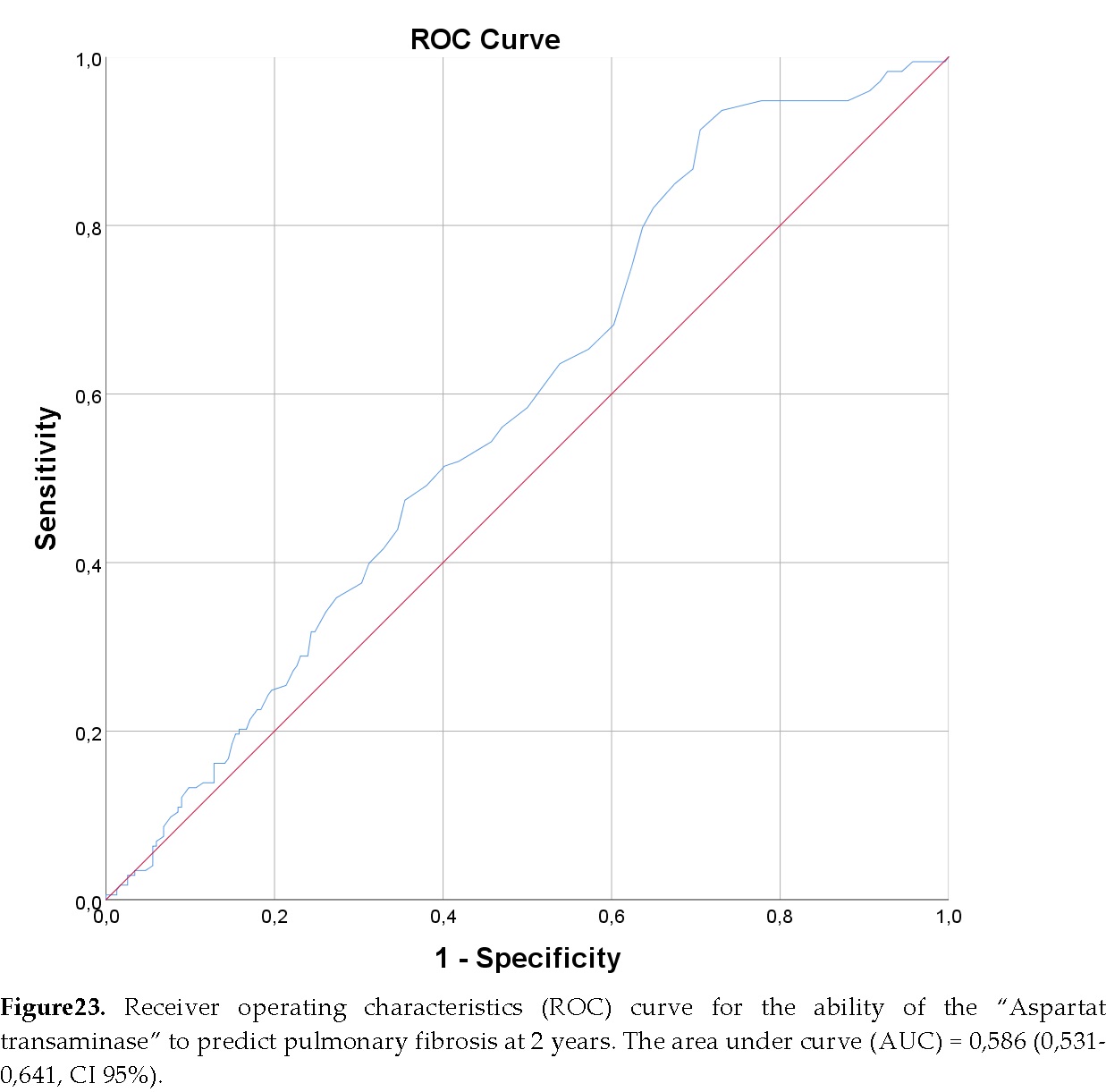

Supplement: Supplementary file 1 [file diagnostics-14-02811-s001.zip › Figure S23. ROC curve - aspartat transaminase - 2 years fibrosis.jpg]

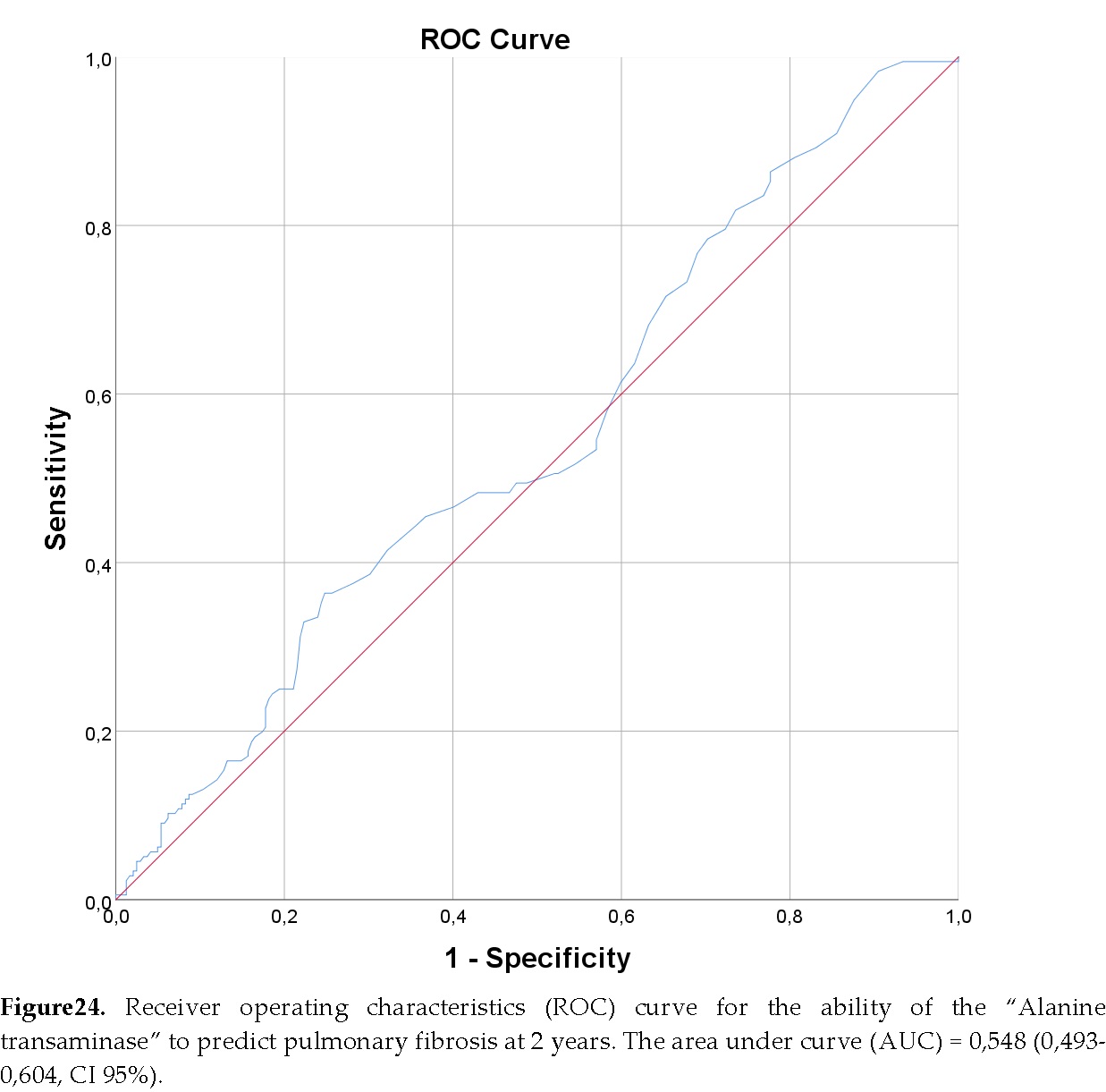

Supplement: Supplementary file 1 [file diagnostics-14-02811-s001.zip › Figure S24. ROC curve - alanine transaminase - 2years fibrosis.jpg]

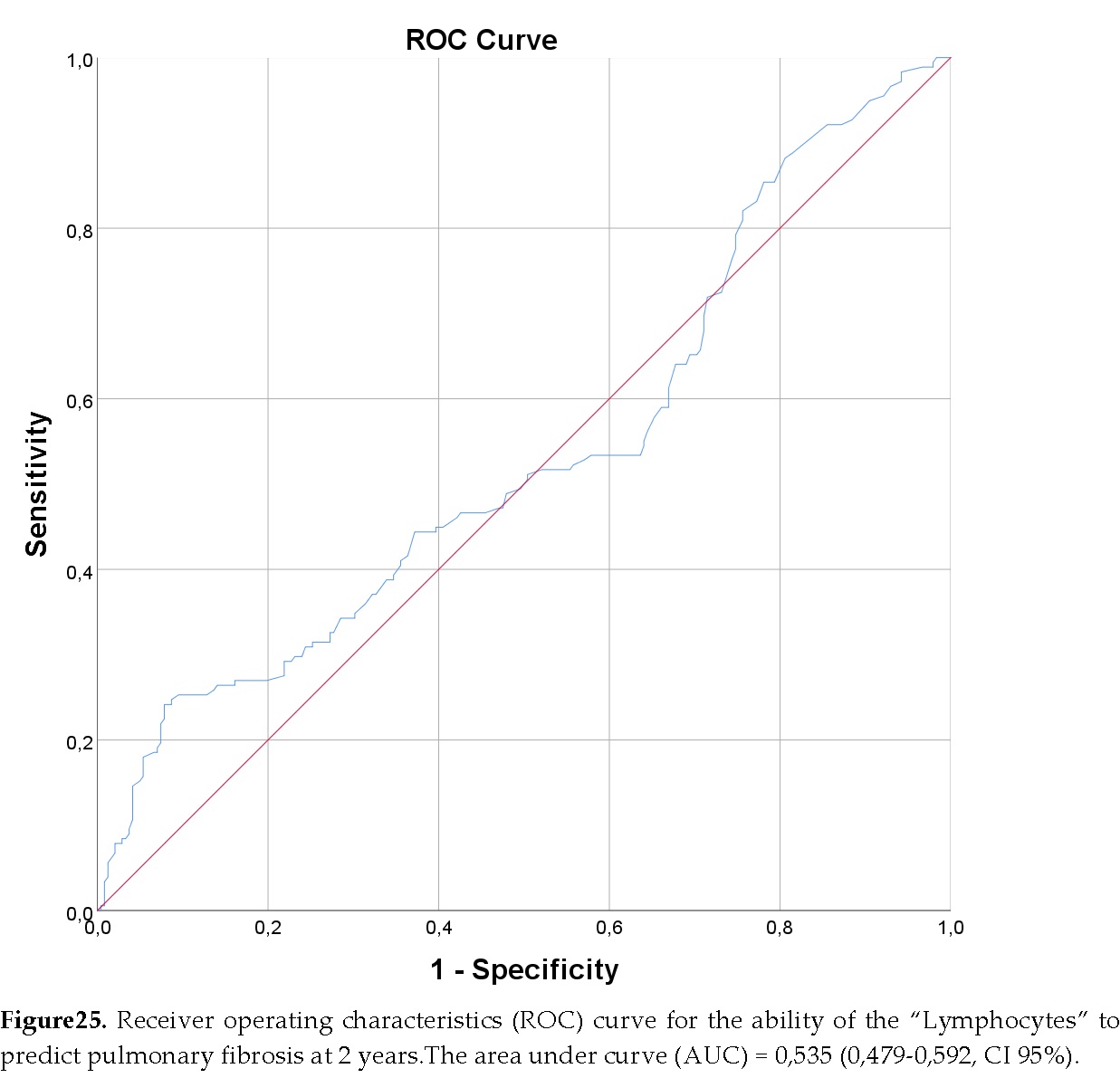

Supplement: Supplementary file 1 [file diagnostics-14-02811-s001.zip › Figure S25. ROC curve - lymphocytes - 2 years fibrosis.jpg]

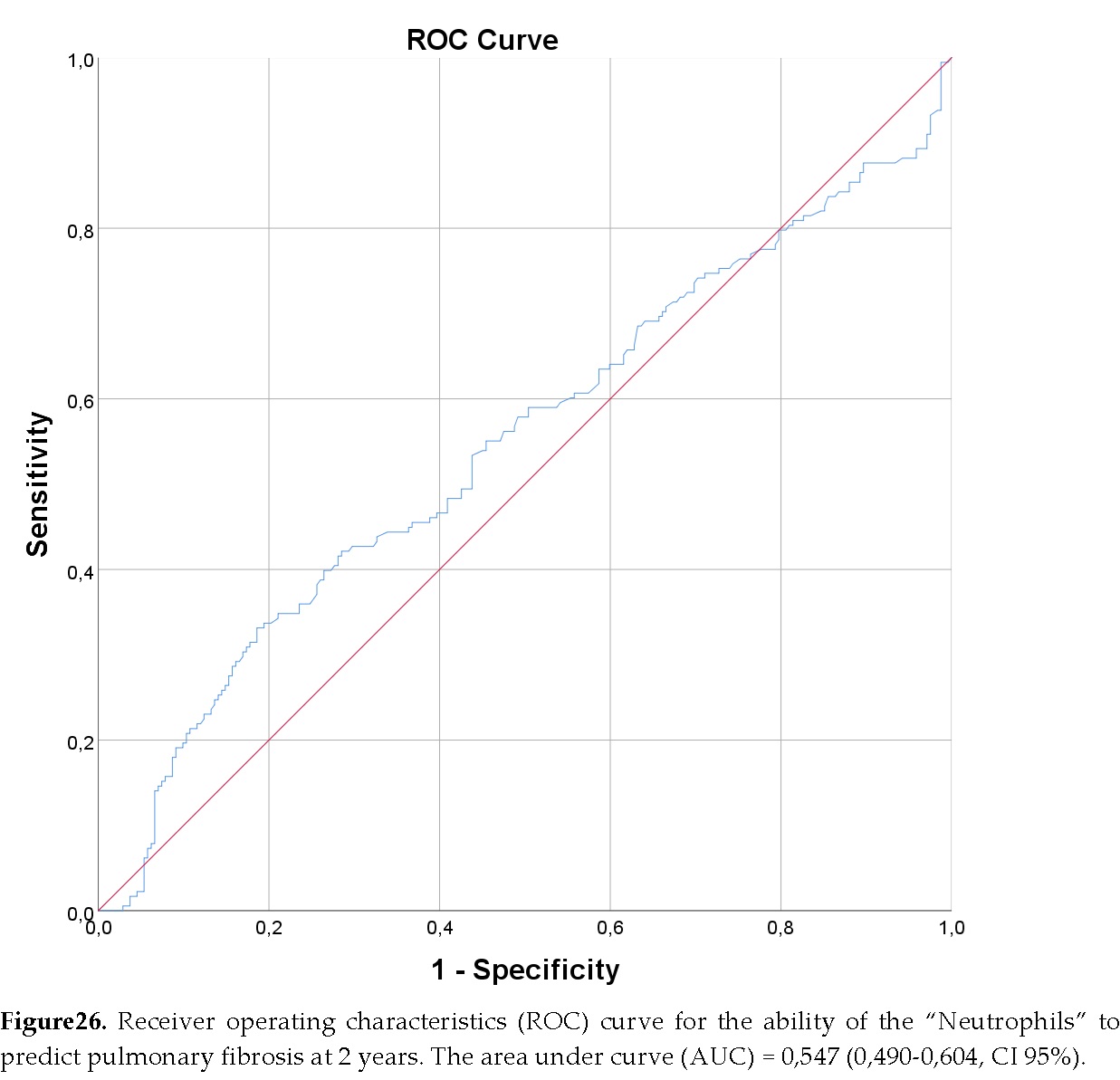

Supplement: Supplementary file 1 [file diagnostics-14-02811-s001.zip › Figure S26. ROC curve - neutrophils - 2 years fibrosis.jpg]

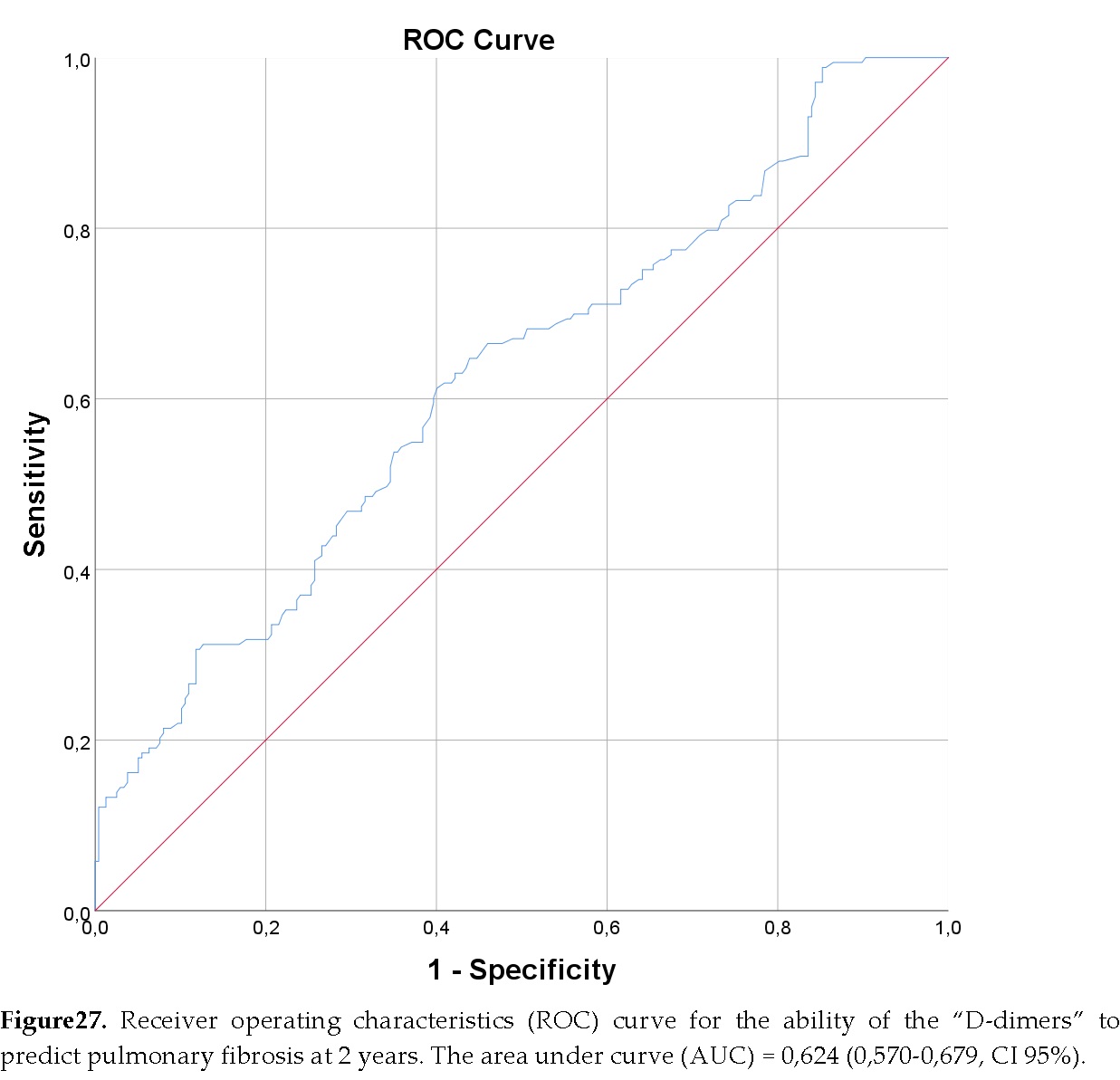

Supplement: Supplementary file 1 [file diagnostics-14-02811-s001.zip › Figure S27. ROC curve - D-dimers - 2 years fibrosis.jpg]

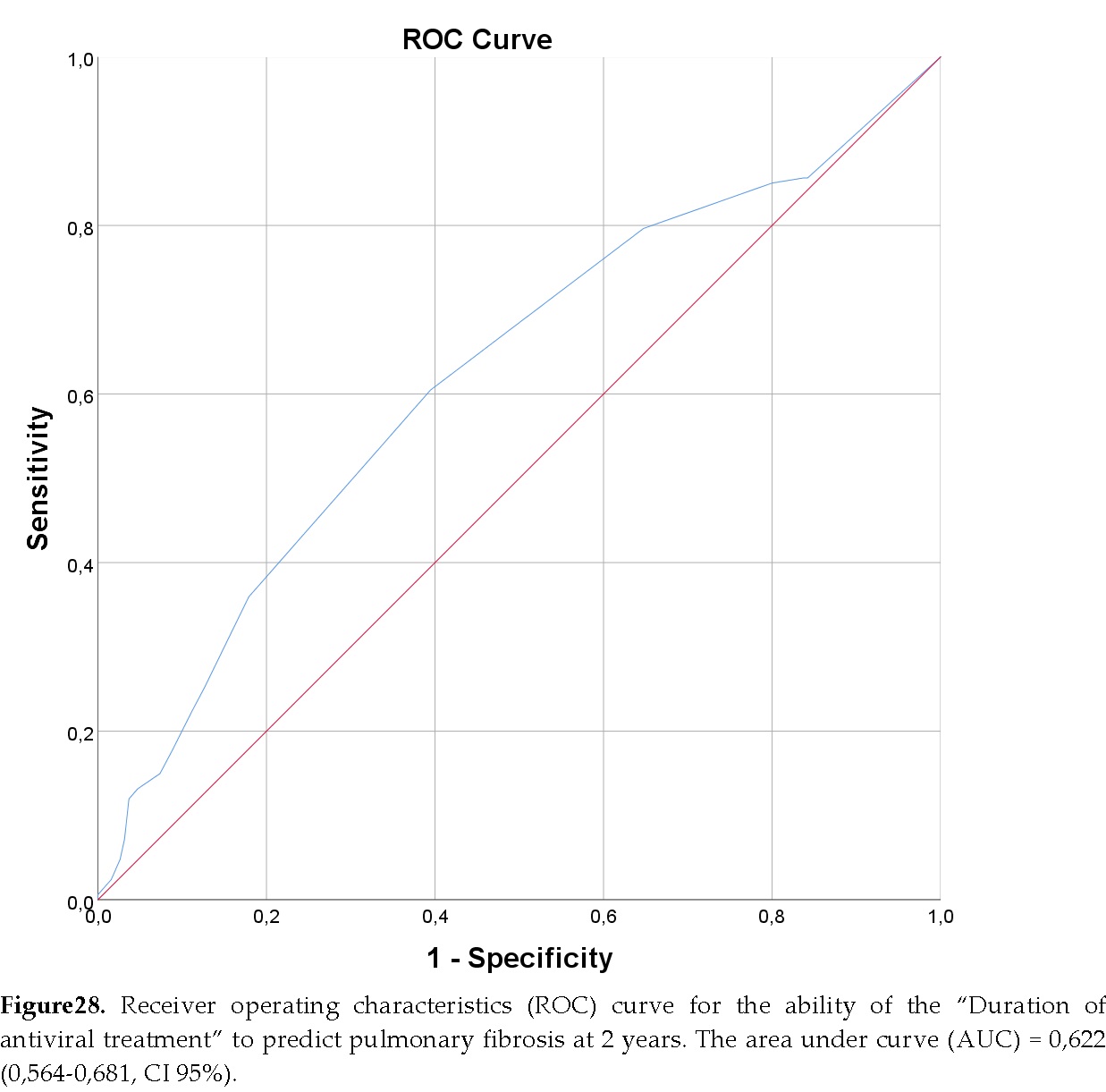

Supplement: Supplementary file 1 [file diagnostics-14-02811-s001.zip › Figure S28. ROC curve - duration of antiviral treatment - 2 years fibrosis.jpg]

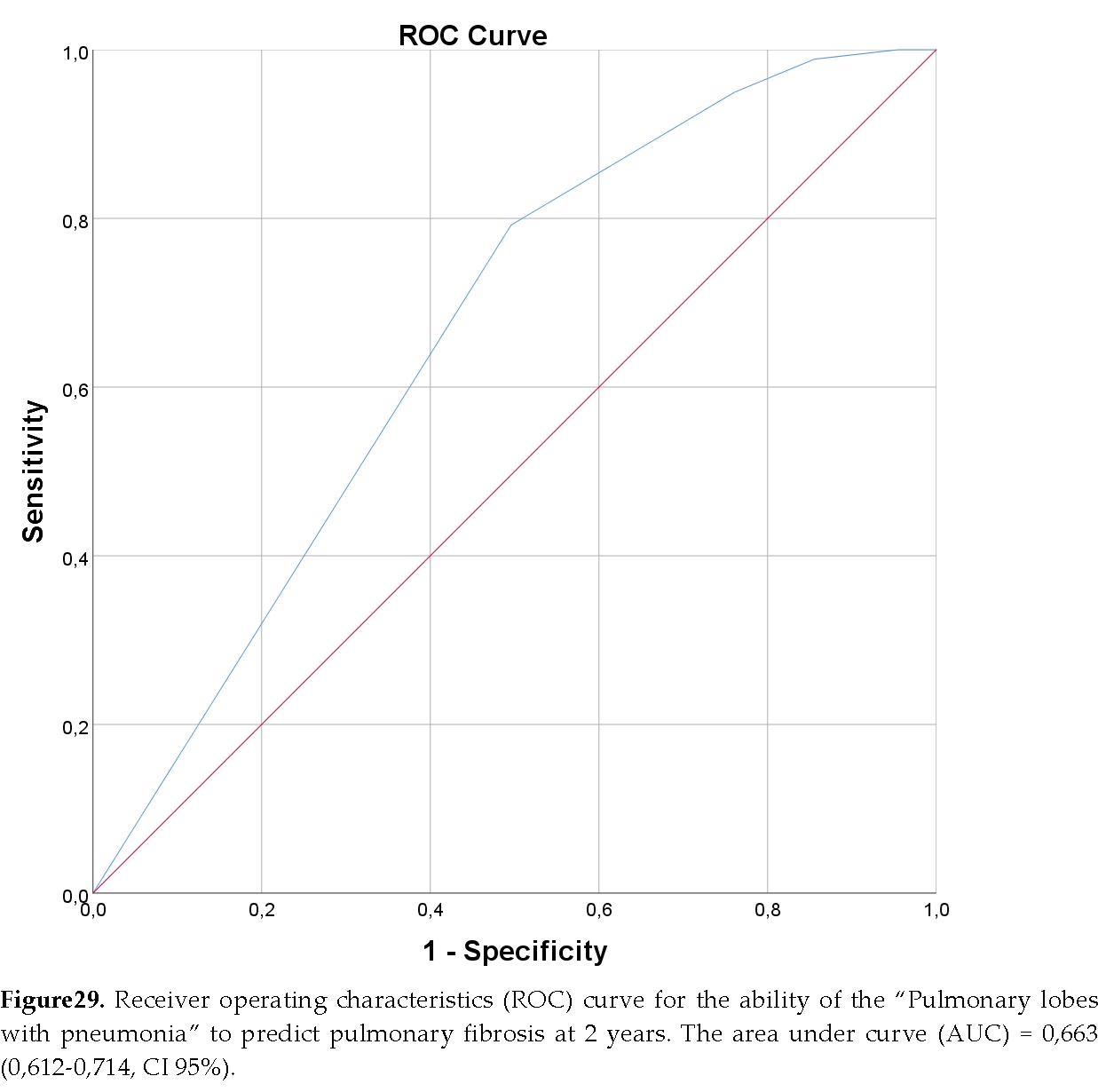

Supplement: Supplementary file 1 [file diagnostics-14-02811-s001.zip › Figure S29. ROC curve - pulmonary lobes with pneumonia - 2 years fibrosis.jpg]

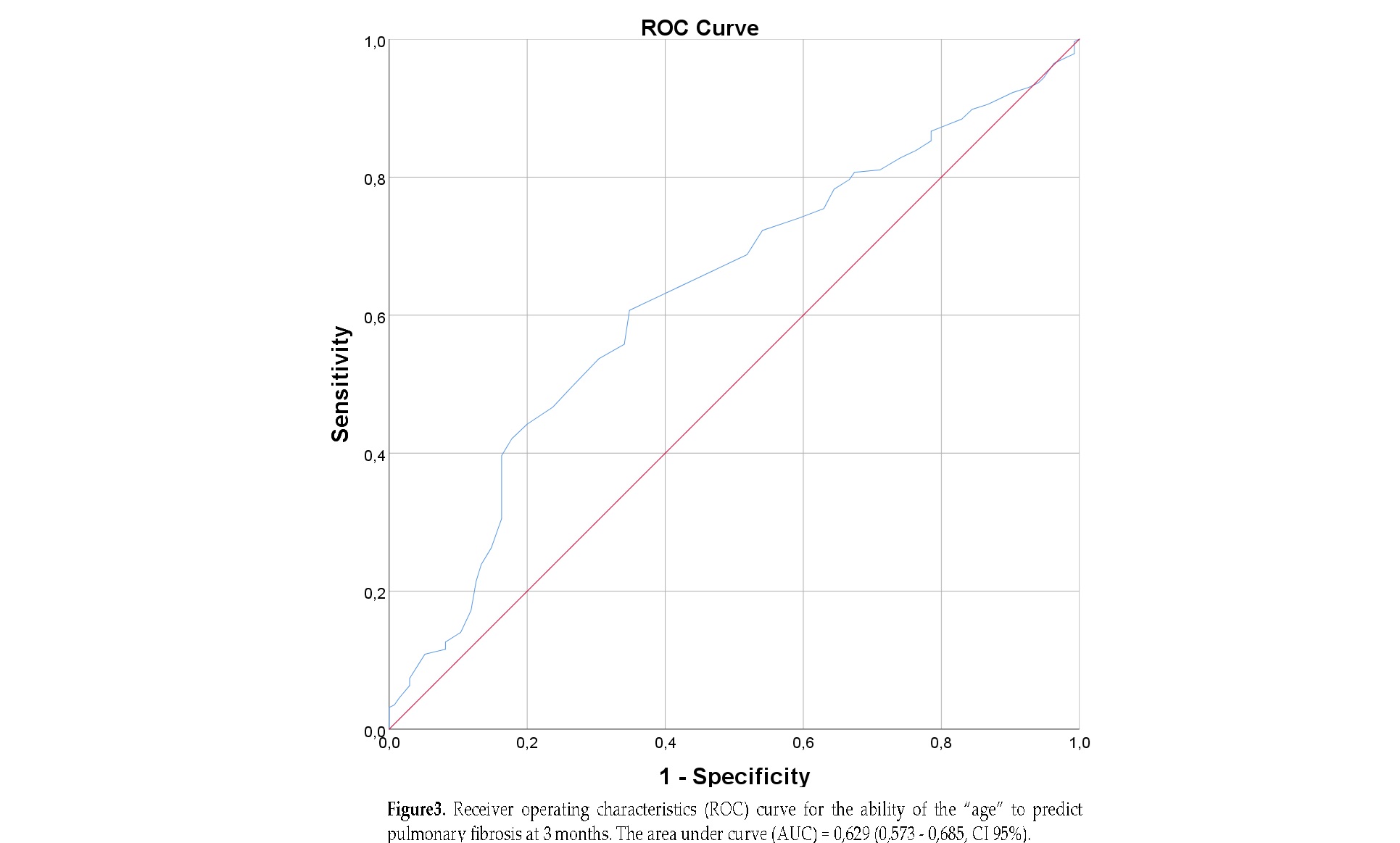

Supplement: Supplementary file 1 [file diagnostics-14-02811-s001.zip › Figure S3. ROC curve - age - 3months fibrosis.jpg]

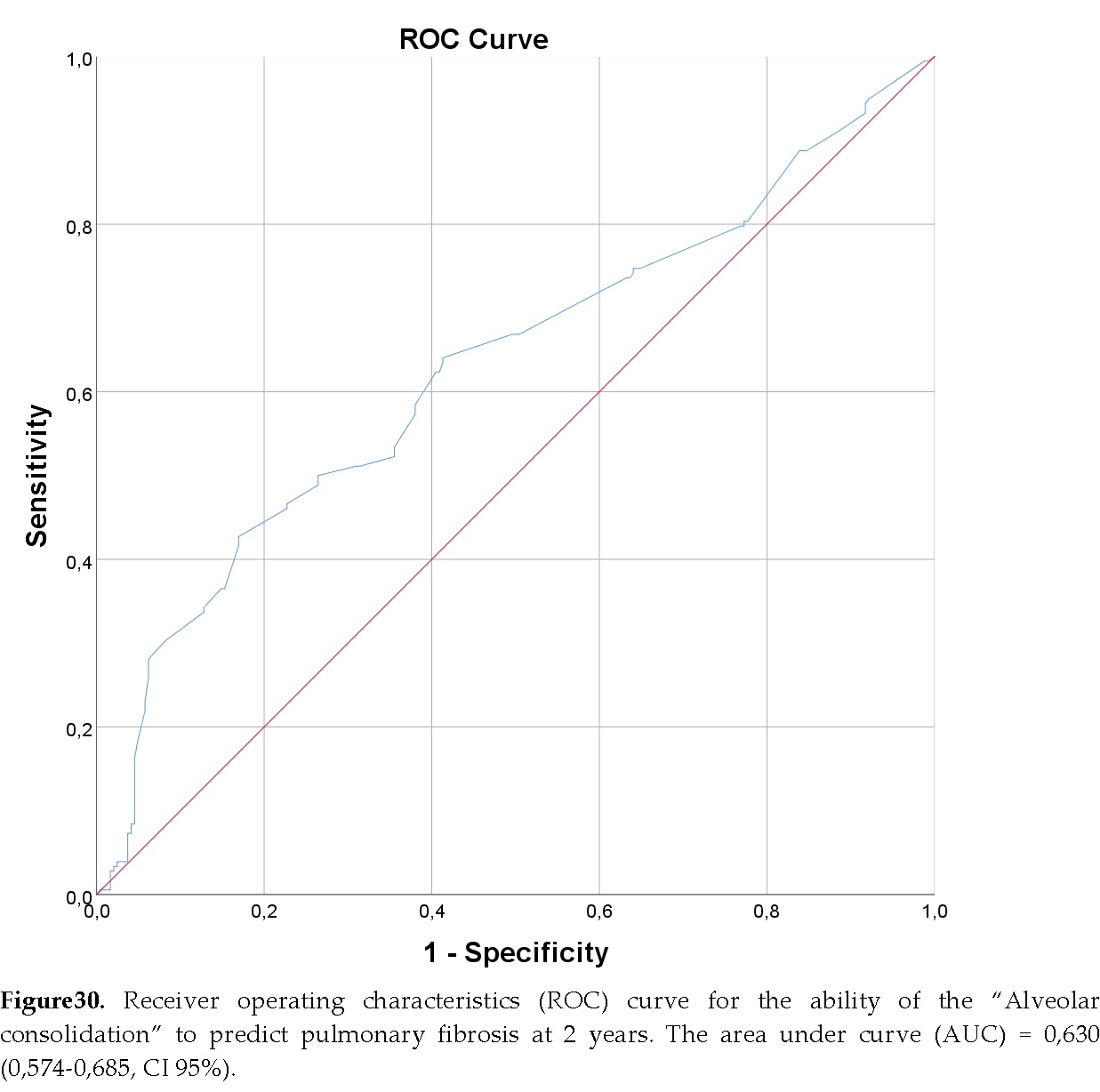

Supplement: Supplementary file 1 [file diagnostics-14-02811-s001.zip › Figure S30. ROC curve - alveolar consolidation - 2 years fibrosis.jpg]

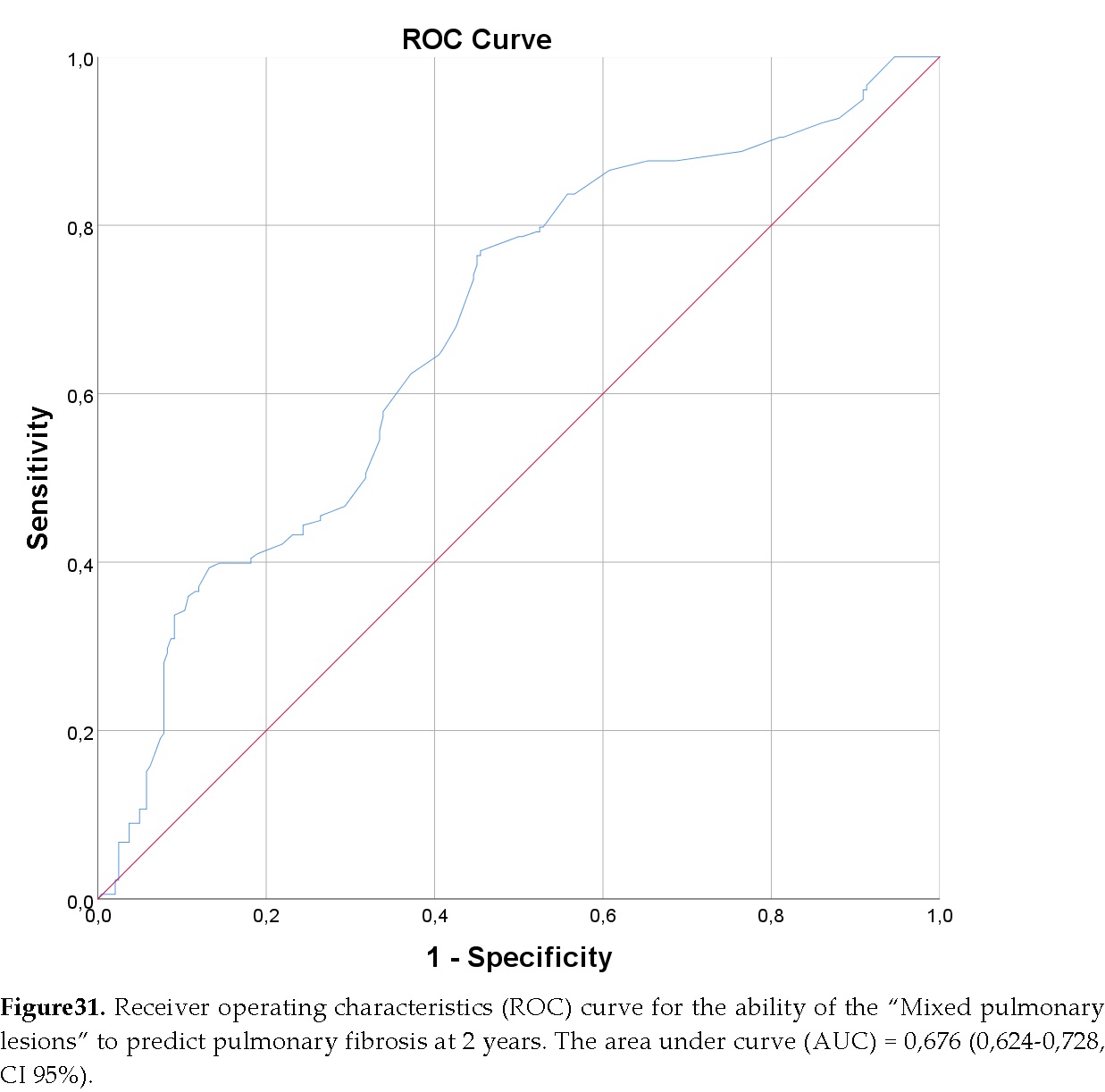

Supplement: Supplementary file 1 [file diagnostics-14-02811-s001.zip › Figure S31. ROC curve - mixed pulmonary lesions - 2 years fibrosis.jpg]

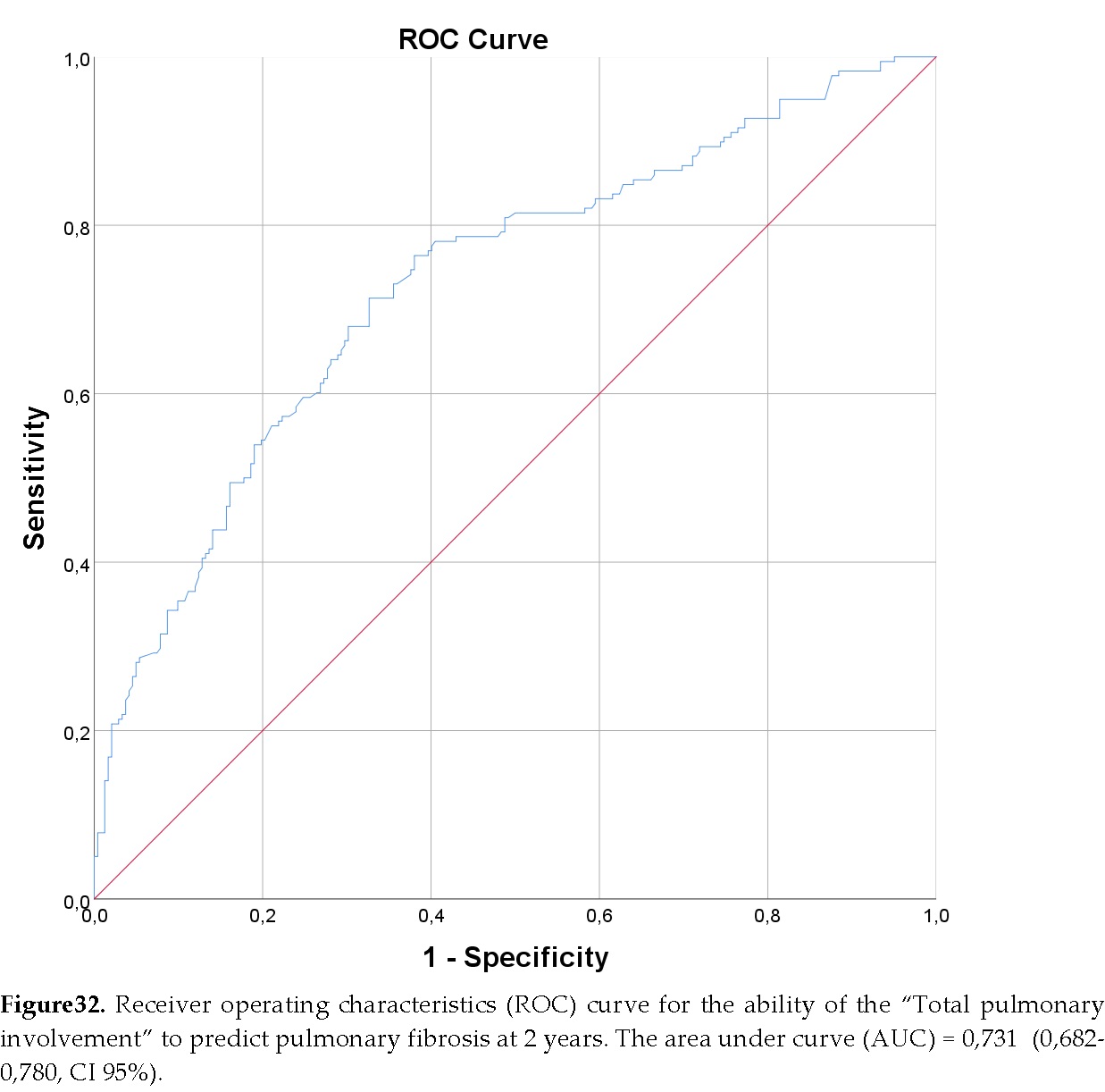

Supplement: Supplementary file 1 [file diagnostics-14-02811-s001.zip › Figure S32. ROC curve - total pulmonary involvement - 2 years fibrosis.jpg]

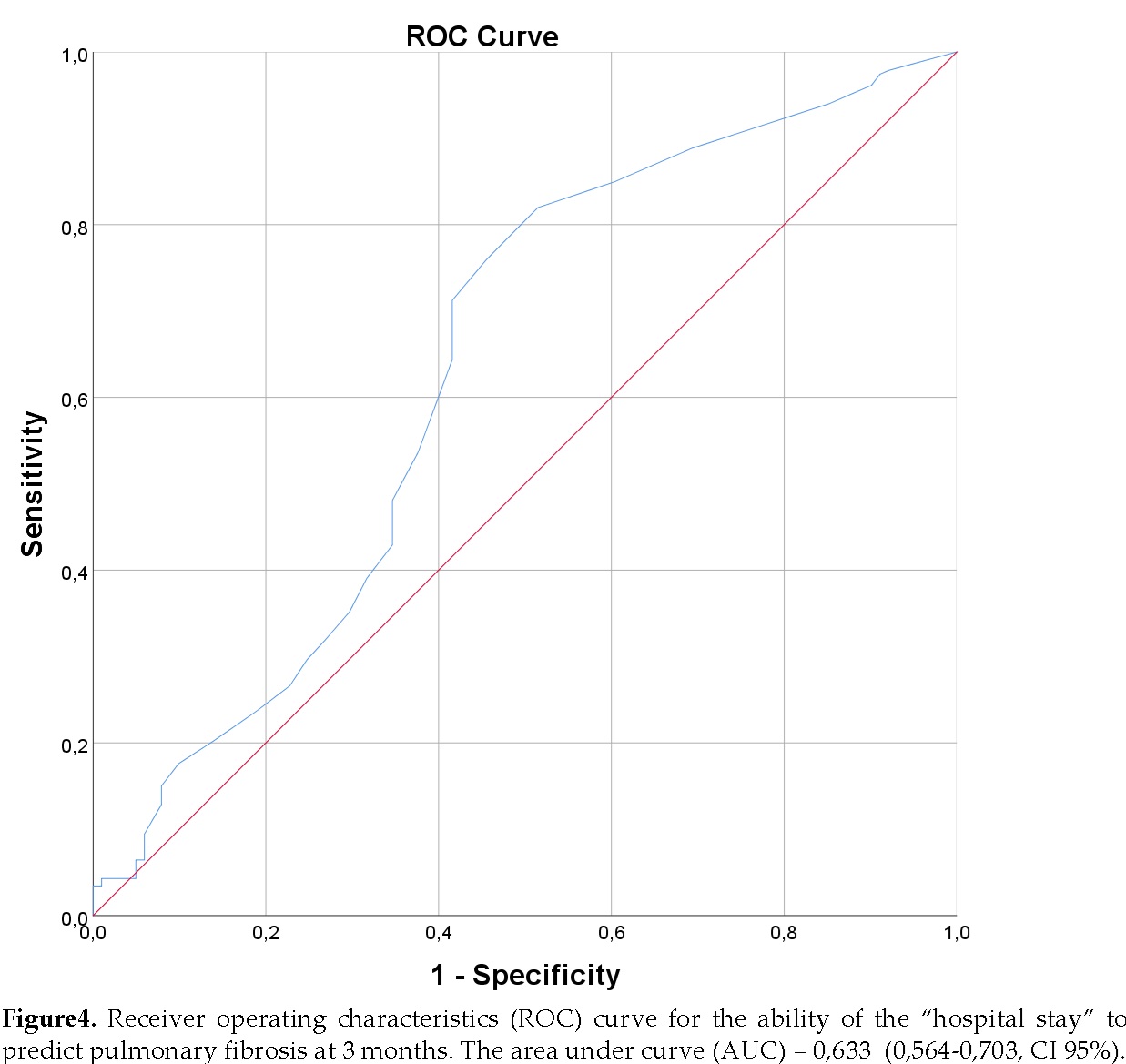

Supplement: Supplementary file 1 [file diagnostics-14-02811-s001.zip › Figure S4. ROC curve - hospital stay - 3months fibrosis.jpg]

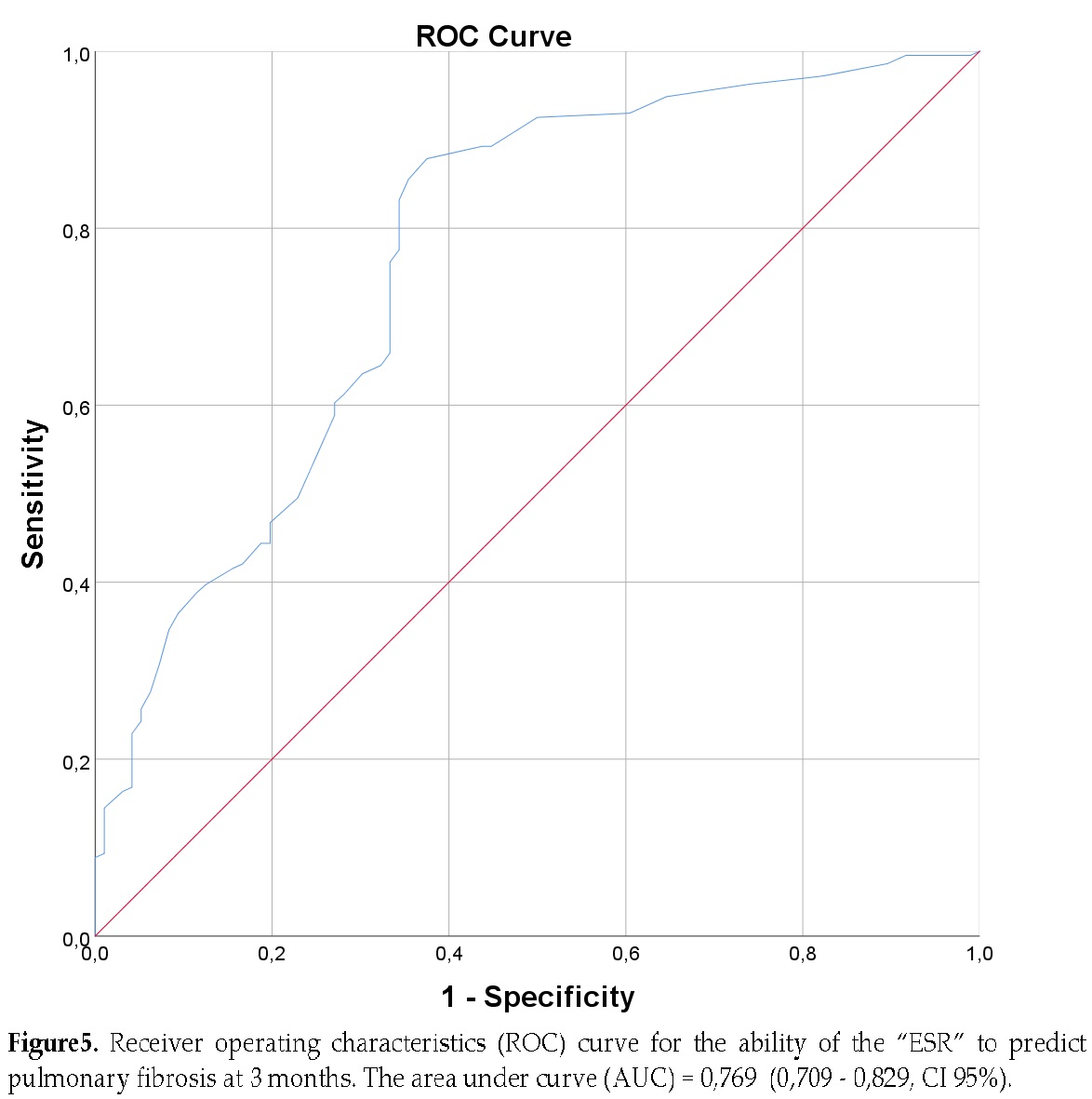

Supplement: Supplementary file 1 [file diagnostics-14-02811-s001.zip › Figure S5. ROC curve - ESR - 3months fibrosis.jpg]

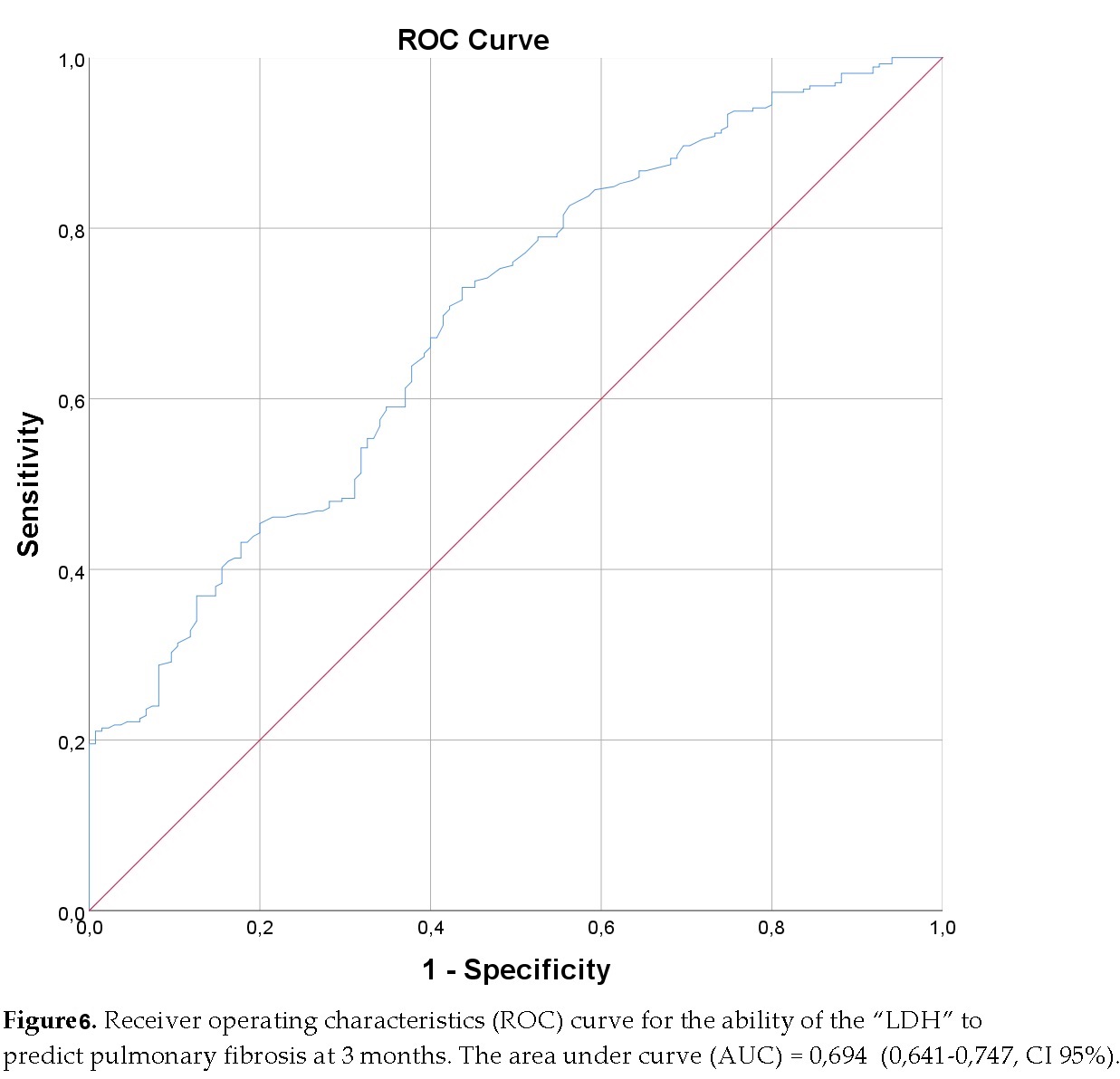

Supplement: Supplementary file 1 [file diagnostics-14-02811-s001.zip › Figure S6. ROC curve - fibrinogen - 3months fibrosis.jpg]

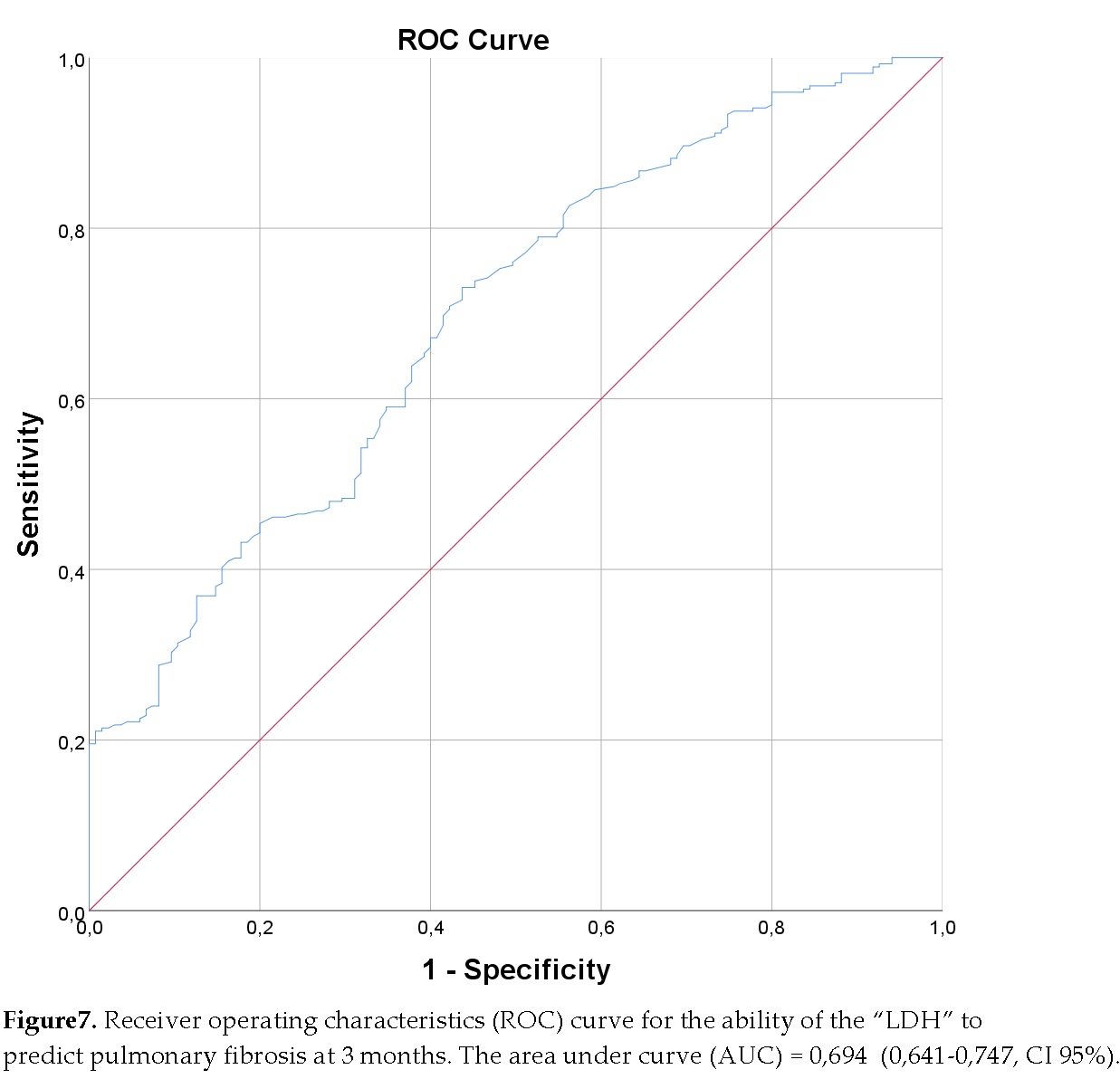

Supplement: Supplementary file 1 [file diagnostics-14-02811-s001.zip › Figure S7. ROC curve - LDH - 3months fibrosis.jpg]

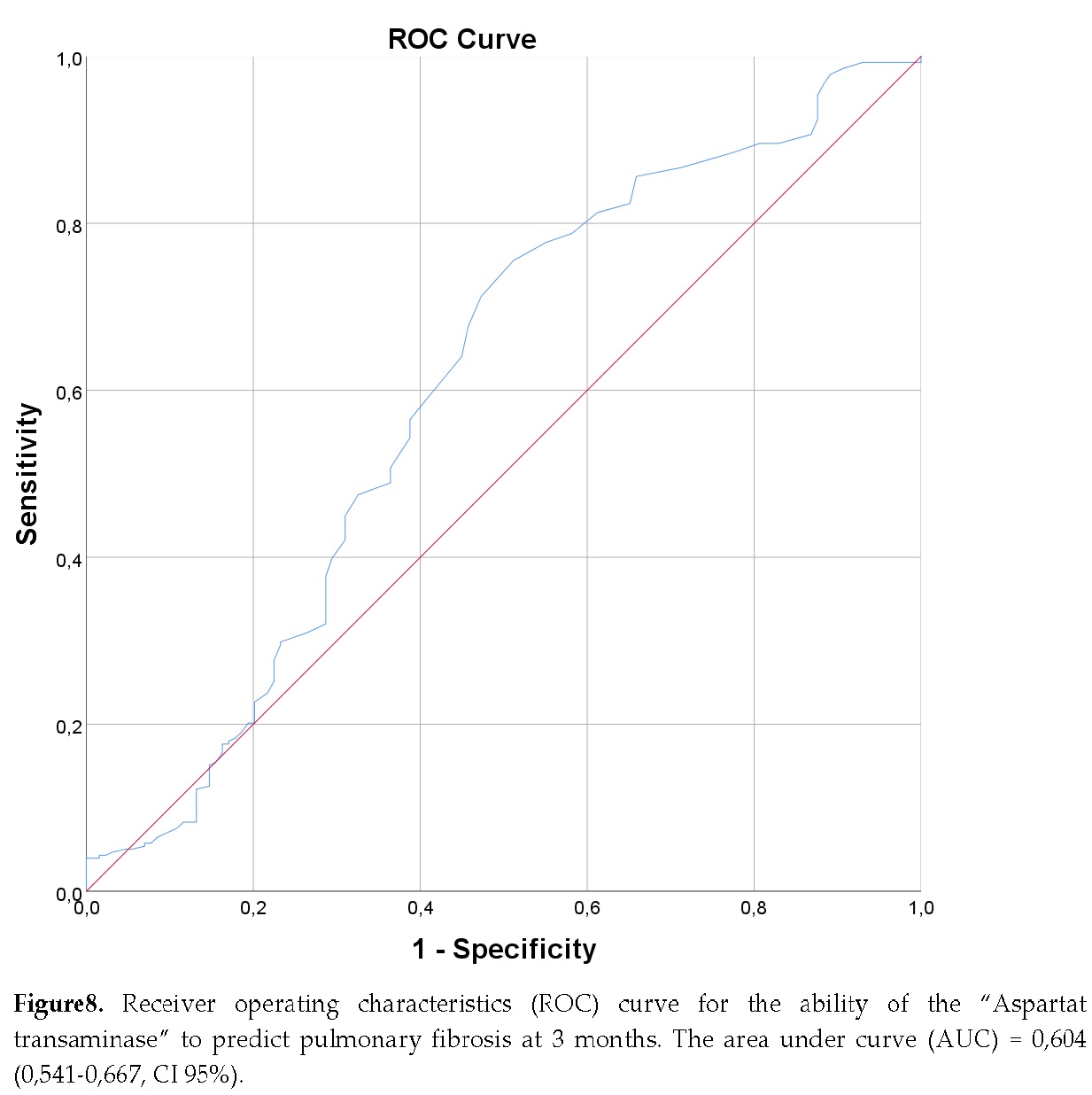

Supplement: Supplementary file 1 [file diagnostics-14-02811-s001.zip › Figure S8. ROC curve - aspartat transaminase - 3months fibrosis.jpg]

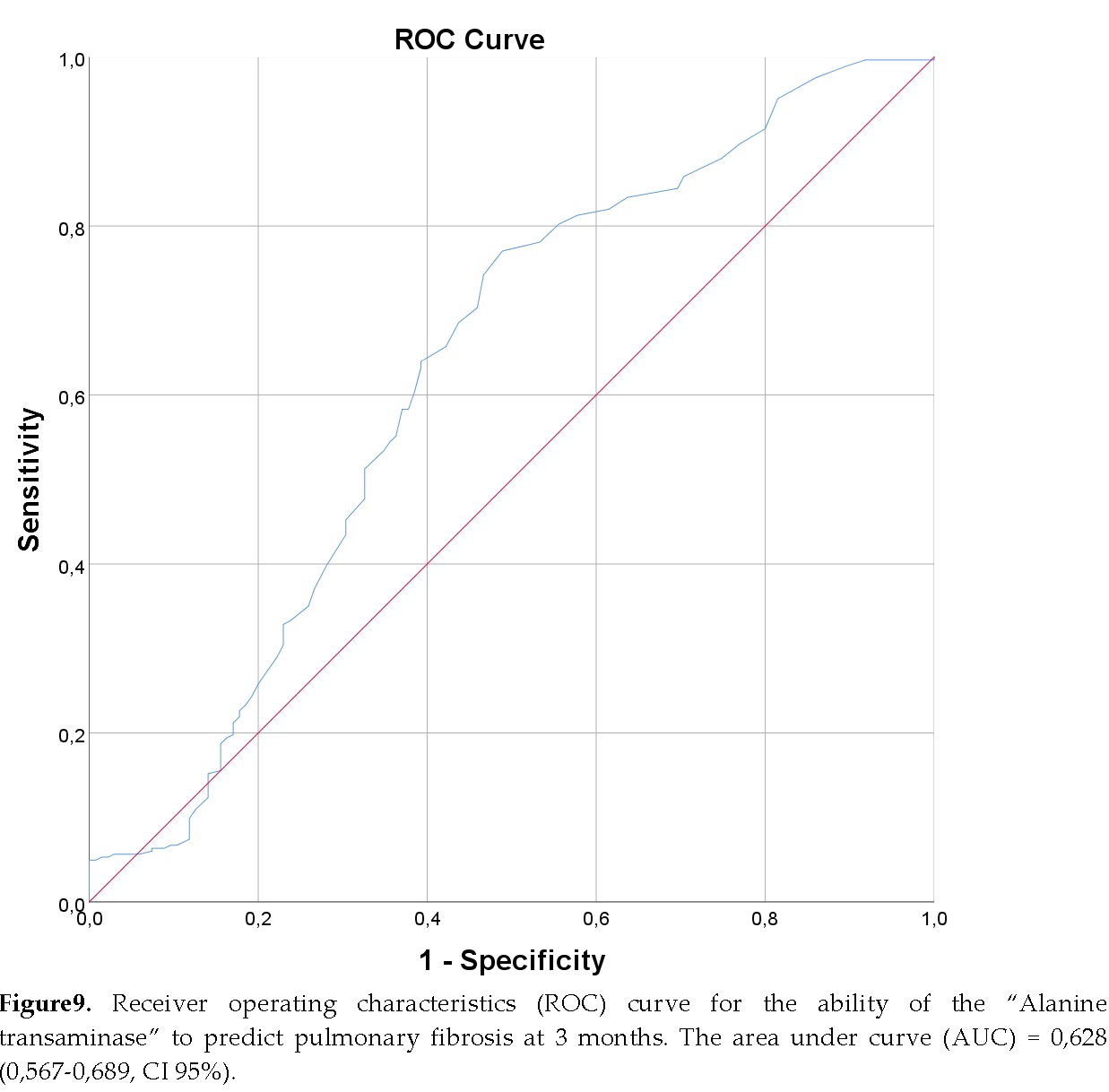

Supplement: Supplementary file 1 [file diagnostics-14-02811-s001.zip › Figure S9. ROC curve - alanine transaminase - 3months fibrosis.jpg]
